# The Histone Demethylase Jarid1b Ensures Faithful Mouse Development by Protecting Developmental Genes from Aberrant H3K4me3

**DOI:** 10.1371/journal.pgen.1003461

**Published:** 2013-04-18

**Authors:** Mareike Albert, Sandra U. Schmitz, Susanne M. Kooistra, Martina Malatesta, Cristina Morales Torres, Jens C. Rekling, Jens V. Johansen, Iratxe Abarrategui, Kristian Helin

**Affiliations:** 1 Biotech Research and Innovation Centre (BRIC), University of Copenhagen, Copenhagen, Denmark; 2 Centre for Epigenetics, University of Copenhagen, Copenhagen, Denmark; 3 Cellular and Systems Neurobiology Laboratory, Department of Neuroscience and Pharmacology, Panum Institute, University of Copenhagen, Copenhagen, Denmark; 4 The Danish Stem Cell Cener (DanStem), University of Copenhagen, Copenhagen, Denmark; University of Pennsylvania, United States of America

## Abstract

Embryonic development is tightly regulated by transcription factors and chromatin-associated proteins. H3K4me3 is associated with active transcription and H3K27me3 with gene repression, while the combination of both keeps genes required for development in a plastic state. Here we show that deletion of the H3K4me2/3 histone demethylase *Jarid1b* (Kdm5b/Plu1) results in major neonatal lethality due to respiratory failure. *Jarid1b* knockout embryos have several neural defects including disorganized cranial nerves, defects in eye development, and increased incidences of exencephaly. Moreover, in line with an overlap of Jarid1b and Polycomb target genes, *Jarid1b* knockout embryos display homeotic skeletal transformations typical for Polycomb mutants, supporting a functional interplay between Polycomb proteins and Jarid1b. To understand how Jarid1b regulates mouse development, we performed a genome-wide analysis of histone modifications, which demonstrated that normally inactive genes encoding developmental regulators acquire aberrant H3K4me3 during early embryogenesis in *Jarid1b* knockout embryos. H3K4me3 accumulates as embryonic development proceeds, leading to increased expression of neural master regulators like *Pax6* and *Otx2* in *Jarid1b* knockout brains. Taken together, these results suggest that Jarid1b regulates mouse development by protecting developmental genes from inappropriate acquisition of active histone modifications.

## Author Summary

Histone modifications are involved in transcriptional regulation and thus affect cellular identity, differentiation, and development. We study the histone demethylase Jarid1b (Kdm5b/Plu1), as it has been reported to be highly expressed in several human cancers and therefore might present a novel target for anti-cancer therapies. To gain insights into the physiological role of Jarid1b, we have generated a *Jarid1b* knockout mouse. We show that loss of Jarid1b affects survival of newborn mice and that Jarid1b is required for the faithful development of several neural organs. To understand how Jarid1b regulates embryogenesis, we identified genes with increased H3K4me3 at a genome-wide scale as well as Jarid1b target genes during development. In *Jarid1b* knockout embryos, master regulators of neural development are expressed at higher levels, underscoring the importance of Jarid1b in transcriptional regulation. Furthermore, we extend previous reports of overlapping Jarid1b and Polycomb target genes to show the functional relevance of this observation. Our results provide the first detailed analysis of the role of Jarid1b in normal development and provide a basis for further studies evaluating the contribution of Jarid1b to tumorigenesis.

## Introduction

Embryonic development is characterized by a coordinated program of proliferation and differentiation that is tightly regulated by transcription factors and chromatin-associated proteins. As embryonic cells differentiate, certain genes are activated while others are repressed, resulting in a unique pattern of gene expression in each cell type.

Histone H3 lysine 4 tri-methylation (H3K4me3) localizes to transcription start sites with high levels present at actively transcribed genes [1], [2], even though H3K4me3 at promoters is not a definite indication for transcriptional activity [3]. Methylation of H3K4 is catalyzed by a family of 10 histone methyltransferases in mammals [4]. Five of these are members of the Trithorax group of proteins that were first described in *Drosophila* to be required for maintenance of *Hox* gene expression by counteracting Polycomb-mediated repression. In *Mll1* and *Mll2* mutant mice, target genes are properly activated but expression fails to be maintained leading to embryonic lethality [5], [6]. In addition, H3K4 histone methyltransferases function in hematopoiesis [7], [8] and neurogenesis [9].

H3K4me3 is found in a constant balance with Polycomb-mediated repressive H3K27me3. Presence of both H3K4me3 and H3K27me3 at promoters is referred to as bivalency [10]. The category of bivalent genes is enriched in developmental regulators and is particularly abundant in embryonic stem cells (ESCs) that have the potential for several lineage choices [11]. Moreover, Polycomb proteins repress non-lineage specific gene expression, thereby ensuring developmental potency of embryonic and tissue stem cells during lineage specification, differentiation and development (reviewed in [12]). Polycomb proteins are classified into two separate complexes referred to as Polycomb repressive complex 2 (PRC2), which mediates H3K27me3, and PRC1, which catalyzes mono-ubiquitylation of H2A (H2AK119ub1) [13], [14]. Classical models propose a sequential mechanism in which H3K27me3 creates a binding site for PRC1 leading to further repression [14], [15], even though emerging studies suggest that Polycomb function is more complex [16]–[18].

While histone methylation was initially viewed as a stable modification, the discovery of histone demethylating enzymes has changed this paradigm [19]. Demethylation of H3K4me3 is catalyzed by the JARID1 (KDM5) family, which in mammals has four members: JARID1A, JARID1B, JARID1C and JARID1D [20]. The *Drosophila* JARID1 homologue LID (Little imaginal discs) is required for normal development [21], and the *C. elegans* homologue RBR-2 (retinoblastoma binding protein related 2) regulates vulva formation and lifespan [22], [23]. Mice mutant for *Jarid1a* are viable, displaying only mild phenotypes in hematopoiesis and behavior [24]. A recent report suggests that *Jarid1b* mutant mice are embryonic lethal between E4.5 and E7.5 [25]. The molecular mechanisms underlying this phenotype were not addressed. In contrast, others obtained viable *Jarid1b* mutant mice [26]. However, the requirement of Jarid1b for the differentiation of ESCs along the neural lineage [27], [28] suggests that Jarid1b may function in mouse development. In humans, JARID1B is highly expressed in several types of cancer, and it was shown to regulate proliferation of breast cancer cells and a slow cycling population of melanoma cells that promotes prolonged tumor growth (reviewed in [20]).

While the role of *Jarid1b* in mice remains controversial [25], [26], an understanding of its *in vivo* function is essential to direct future studies evaluating JARID1B as a potential drug target in cancer therapy. Jarid1b expression has been reported in various tissues during mouse embryogenesis whereas its expression becomes restricted in adults [29]. Here we report the first detailed analysis of the contribution of *Jarid1b* to mouse development. We show that *Jarid1b* is required for the proper development of several neural systems in the mouse and address the mechanisms underlying the observed defects.

## Results

### Knockout of *Jarid1b* leads to post-natal lethality in the majority of pups

**Figure 1:**
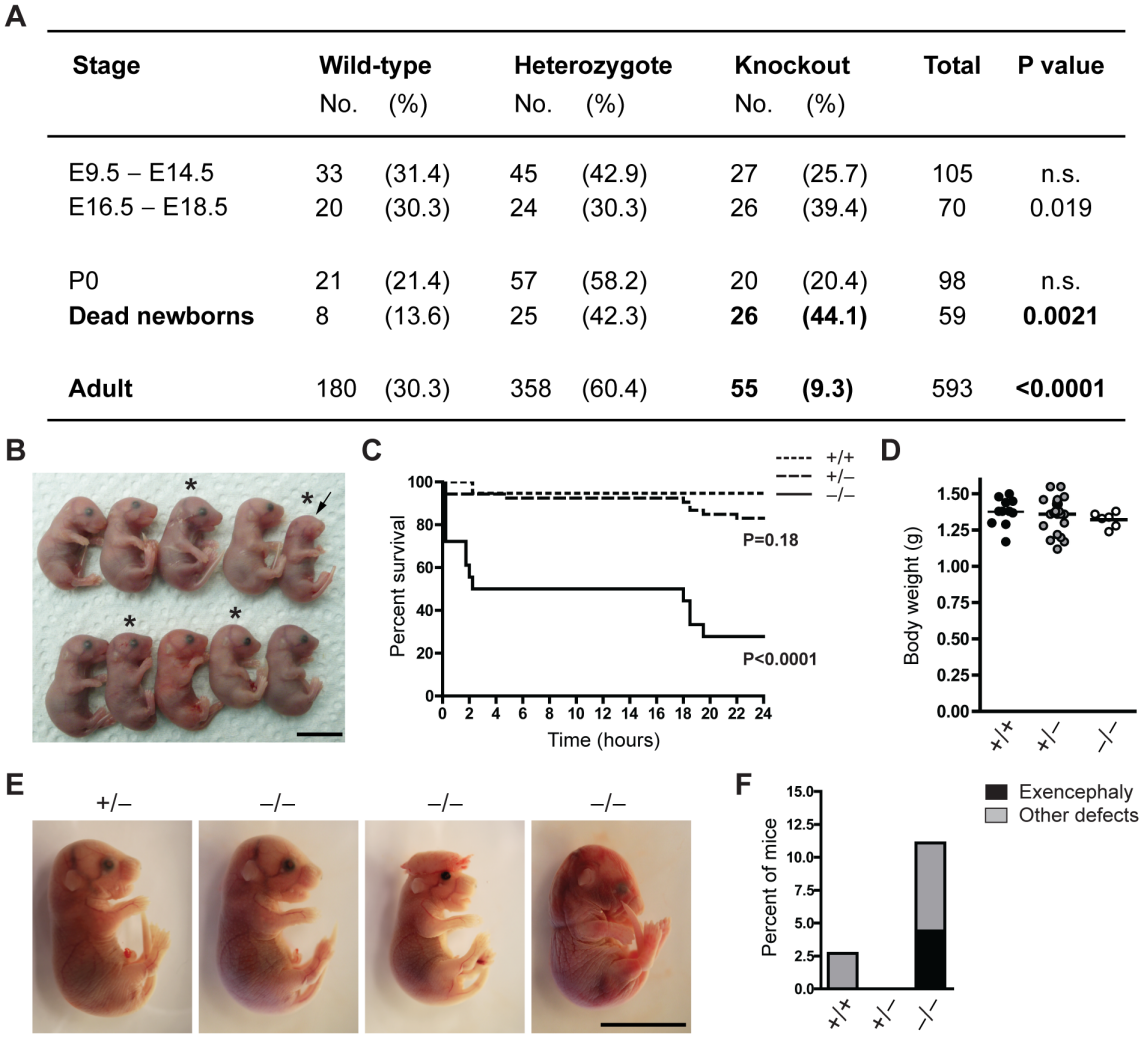
The majority of Jarid1b knockouts die within the first day after birth. (*A*) The number of *Jarid1b* wild-type, heterozygous and knockout mice is presented for the indicated stages. P0 refers to pups immediately after caesarian delivery. Dead newborns are pups that were found dead after natural birth (total number of pups examined n = 649; Note that dead pups are often cannibalized by the mothers, and thus the numbers present an underestimation). P values were calculated using a Chi-square test. (*B*) Litter obtained from *Jarid1b* heterozygotes immediately after caesarean delivery. Asterics indicate *Jarid1b* knockout pups. Note that the *Jarid1b* knockout pup marked by an arrow lacks eyes. (*C*) Survival curve of *Jarid1b* wild-type (n = 19), heterozygous (n = 54) and knockout (n = 18) pups during the first day after caesarean delivery. (*D*) Body weight of newborns. (*E*) Examples of E18.5 embryos with indicated genotypes. While the left knockout embryo appears normal, the second shows exencephaly while the third one has heterogeneous defects. (*F*) Frequency of *Jarid1b* wild-type (n = 37), heterozygous (n = 83) and knockout (n = 50) pups and late embryos that develop exencephaly or other heterogeneous defects. Scale bars, 1 cm.

To characterize the function of Jarid1b during mouse development, we generated constitutive *Jarid1b* knockout mice. Conditionally targeted *Jarid1b* mice containing a lacZ-Neo-reporter cassette flanked by *FRT* sites and in which *Jarid1b* exon 6 is flanked by *loxP* sites [28] were crossed with mice constitutively expressing *Flp* and *Cre* recombinase to obtain *Jarid1b* ^+/−^ mice. *Jarid1b* ^+/−^ mice were further intercrossed to generate *Jarid1b* ^−/−^ mice. Instead of the expected 25 percent of knockout mice, we only obtained 9.3 percent of adult *Jarid1b* knockouts (Figure 1A), suggesting that *Jarid1b* ^−/−^ mice are sub-viable. Analysis of early and late embryos from *Jarid1b* ^+/−^ intercrosses showed expected ratios while an increased number of *Jarid1b* knockouts was present among pups found dead during the first day after birth (Figure 1A), indicating that this might be the critical time for survival.

We have previously shown that conditional deletion of *Jarid1b* using this construct *in vitro* results in complete loss of Jarid1b protein and no generation of truncated or alternatively spliced variants [28]. Loss of *Jarid1b in vivo* was confirmed in all *Jarid1b* ^−/−^ embryos tested (see examples in Figure S1A and S1B), indicating that partial survival of *Jarid1b* knockouts in not due to incomplete deletion. Moreover, expression of other Jarid1 family members is unchanged both *in vitro* [28] and *in vivo* (Figure S1C).

To determine more precisely when *Jarid1b* ^−/−^ pups die, we performed caesarean deliveries and closely monitored the pups (Figure 1B). While approximately 95 percent of wild-type pups survive, we found that 50 percent of the knockouts die within the first two hours after delivery and another approximately 20 percent die after 14 to 24 hours (Figure 1C). All pups that survive the first day, develop normally until adulthood. Interestingly, survival of *Jarid1b* ^+/−^ pups is also slightly, even though not significantly, reduced during the first day. While most of the *Jarid1b* knockouts are grossly normal and not generally growth retarded (Figure 1D), we observed an increased incidence of developmental defects like exencephaly and eye defects among *Jarid1b* knockouts (Figure 1B, 1E and 1F). Taken together, loss of *Jarid1b* leads to major neonatal lethality of which only a small fraction can be explained by severe morphological abnormalities.

### Respiratory function is not properly established in *Jarid1b* knockout pups

**Figure 2:**
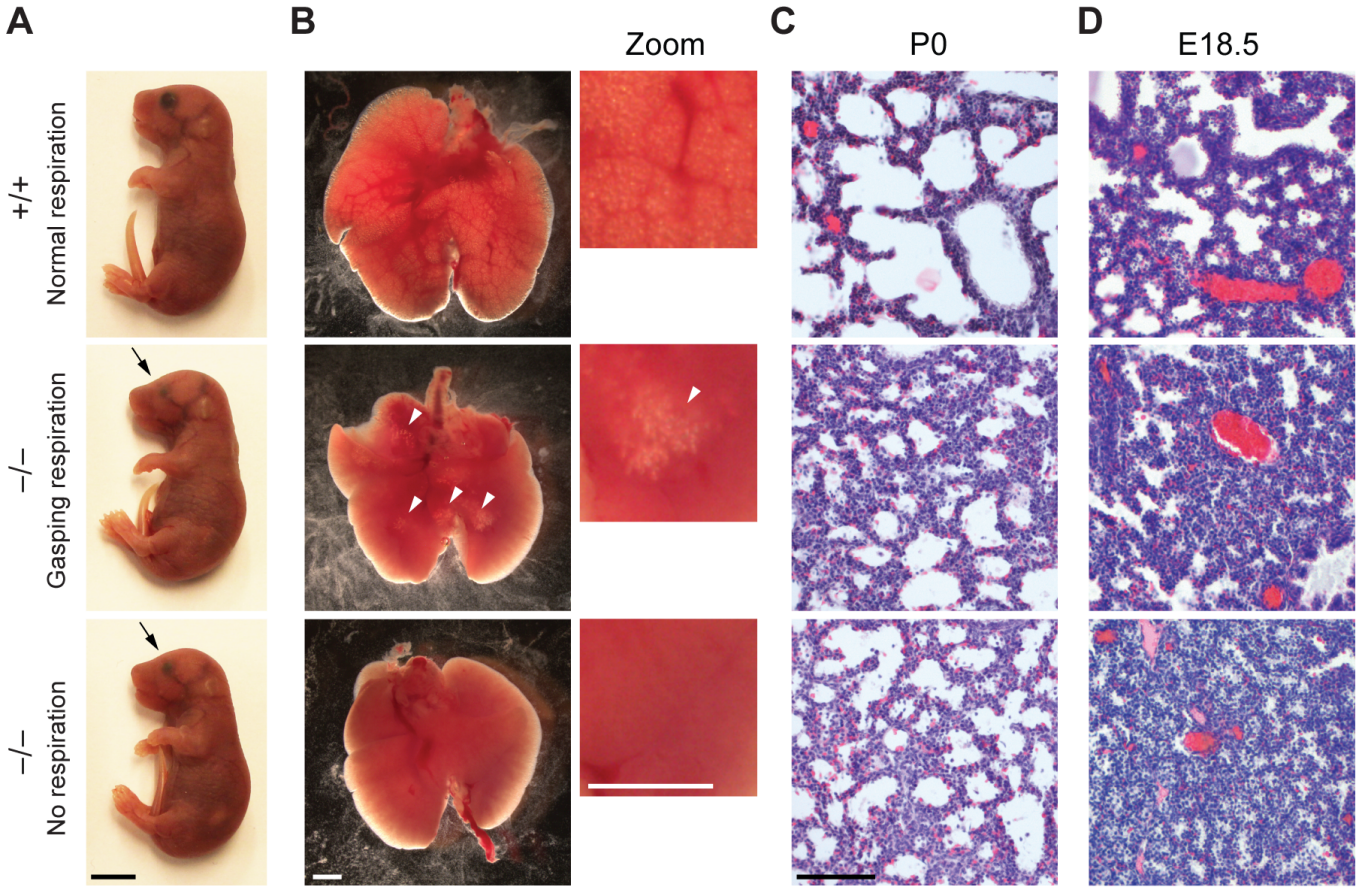
Reduced lung inflation of Jarid1b knockout pups. (*A*) *Jarid1b* wild-type and knockout littermates immediately after caesarian delivery. Both knockout pups died within 2 hours. Note that both knockout pups have smaller eyes (arrows). Scale bar, 0.5 cm. (*B*) Lungs isolated 1–4 hours after delivery. Lung inflation is visible in zoomed images on the right. Note that some regions of the lung are inflated (arrowheads) in the knockout pup that shows gasping respiration, while no inflation is visible in the knockout pup that shows no respiration. Scale bars, 1 mm. (*C*, *D*) Hematoxylin and eosin staining of paraffin-embedded left lungs at P0 (*C*) and E18.5 (*D*). Scale bar, 100 µm.

There is a large spectrum of physiological systems whose defects can challenge neonatal survival including those affecting parturition, breathing, suckling and neonatal homeostasis [30]. The first extrauterine challenge for neonates is breathing and since the majority of *Jarid1b* ^−/−^ pups die immediately after birth, we studied the respiratory system in more detail. Analysis of lungs from E18.5 fetuses revealed a normal size and weight (3.34±0.26 versus 3.45±0.44 percent body weight in heterozygotes versus knockouts, respectively) as well as a normal lobulation pattern (data not shown). Next, we isolated lungs from *Jarid1b* ^−/−^ newborns that had died within 2 hours after delivery and had either not shown any sign of breathing or exhibited gasping respiration (Figure 2A). While the wild-type lung showed saccular inflation, knockout lungs were compact and poorly inflated visible both from gross appearance and histology (Figure 2B and 2C), suggesting that *Jarid1b* ^−/−^ neonates die due to an inability to establish normal breathing. Moreover, preterm (E18.5) *Jarid1b* ^−/−^ lungs were abnormally compact compared to controls (Figure 2D), which might indicate a failure of prenatal breathing activity [31].

Respiratory failure might be caused by delayed lung maturation characterized by reduced surfactant expression [32]. Therefore, we analyzed expression of surfactant proteins (*Sftpa1*, *Sftpb*, *Sftpc* and *Sftpd*) in *Jarid1b* knockout mice (Figure S2A). None of the four surfactants was reduced in *Jarid1b* knockout lungs at E18.5, suggesting that respiratory failure is not due to pulmonary immaturity. In agreement with this, intrauterine administration of dexamethasone, a glucocorticoid that induces fetal lung maturation [33], did not improve survival of *Jarid1b* knockout pups (Figure S2B).

We also examined other physiological systems that are required for neonatal survival including the rib cage, diaphragm, craniofacial appearance and the palate as well as the cardiovascular system [30], but did not detect any abnormalities in the *Jarid1b* knockouts (Figure S3A–S3E). We conclude that while the lungs, skeletal and cardiovascular systems are properly developed, *Jarid1b* ^−/−^ neonates are unable to reliably establish respiratory function.

### Central respiratory rhythmogenesis is intact despite abnormal breathing in knockouts

Immediate breathing after birth is also dependent on brainstem rhythmogenic and pattern forming neural circuits that develop before birth [34]. We therefore isolated brains from neonates after caesarean delivery, but found no gross abnormalities or differences in size of *Jarid1b* ^−/−^ brains compared to controls (Figure S3F and S3G). Essential rhythmogenic networks regulating breathing are located in the brainstem. Therefore, we recorded spontaneous C3–C5 nerve activity in an *in vitro* brainstem-spinal cord preparation from E18.5 embryos. Surprisingly, given the respiratory defects in newborn *Jarid1b* knockouts, central respiratory rhythmogenesis was unperturbed in *Jarid1b* ^−/−^ embryos (Figure S3H).

To monitor neurological reflexes of newborn *Jarid1b* ^−/−^ pups, we tested their response to pinching stimuli [35]. As opposed to control neonates, *Jarid1b* mutants only weakly reacted to a tail pinch (Figure S3I), suggesting that *Jarid1b* newborns show motosensory deficits characterized by hyporesponsiveness.

### Cranial and spinal nerves are disorganized in *Jarid1b* knockout embryos

**Figure 3:**
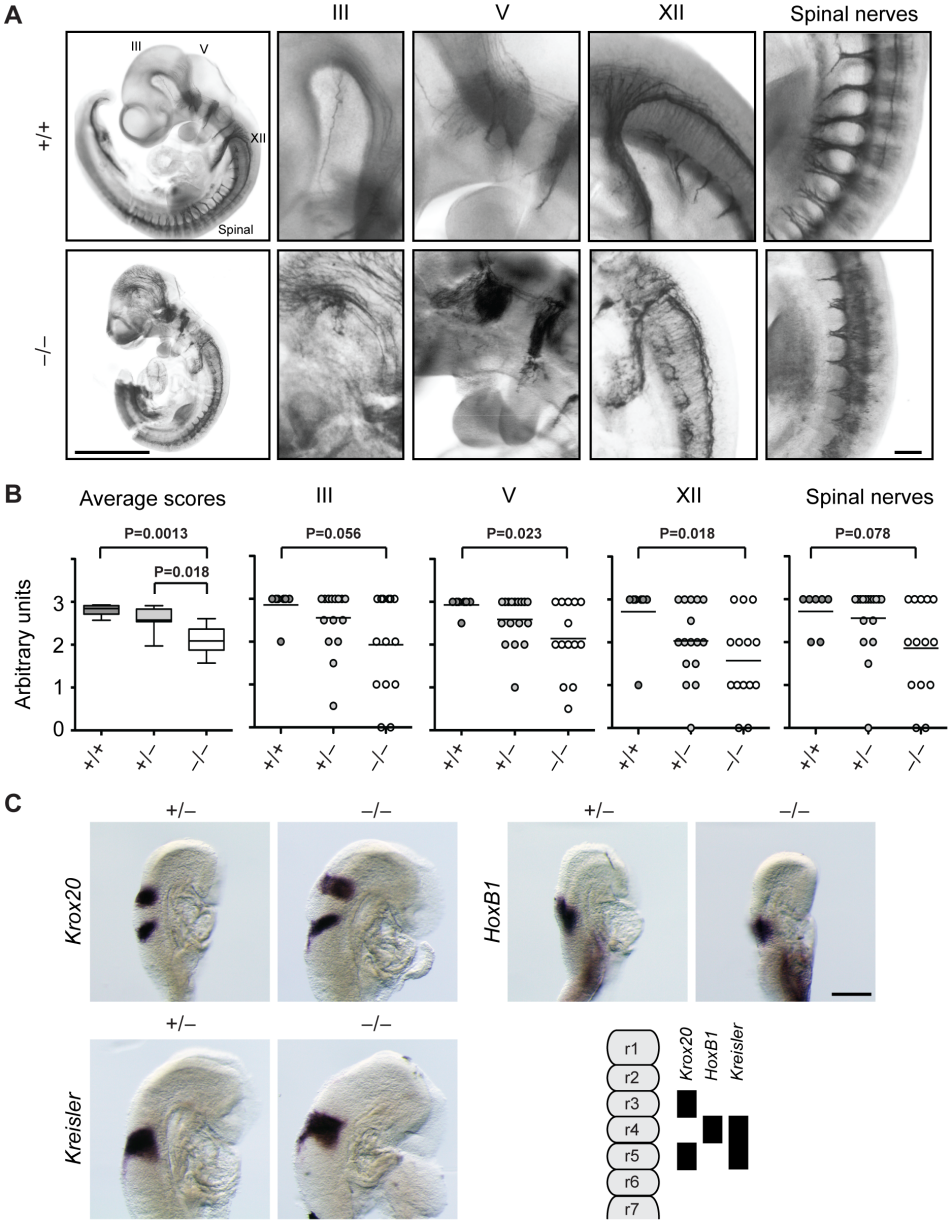
Cranial and spinal nerves are disorganized in Jarid1b knockout embryos. (*A*) Embryos at E10.5 were stained with anti-neurofilament antibody. Shown are examples of a *Jarid1b* wild-type and knockout embryo (scale bar, 1 mm) and zoomed images (scale bar, 100 µm) of cranial nerves III, V and XII and spinal nerves. Note that the tail has been removed from the knockout embryo to facilitate imaging. (*B*) Scoring of nerve integrity: 3 = well defined; 2 = not very distinct, 1 = dysmorphic, 0 = absent. P values were determined by Mann-Whitney test. (*C*) Whole-mount in situ hybridization for *Krox20*, *HoxB1* and *Kreisler* in E8.75 (5–12 somite pairs) *Jarid1b* heterozygous and knockout embryos. Scale bar, 200 µm. A scheme illustrating normal gene expression in the rhombomeric hindbrain is shown at the bottom right.

These results together with our previous *in vitro* data showing that Jarid1b is required for the differentiation of ESCs along the neural lineage [28] prompted us to analyze the development of neural systems in more detail in *Jarid1b* ^−/−^ embryos. As a first step we analyzed cranial nerves, a pair of 12 nerves that are essential for sensory and motor functions and reside in the mid- and hindbrain [36]. Defects in cranial nerve development may compromise neonatal survival. Cranial and spinal nerves can be visualized by whole-mount immunostaining at E10.5 using an anti-neurofilament antibody. Comparison of *Jarid1b* ^−/−^ embryos with controls revealed that while all nerve pairs are present, several cranial and spinal nerves are dysmorphic in the *Jarid1b* knockouts (Figure 3A). We used an arbitrary scoring system to quantify the differences between genotypes and found that *Jarid1b* knockouts are significantly affected while slight defects are already detectable in heterozygotes compared to wild-type (Figure 3B). Cranial nerves are involved in a diverse range of functions including movement of the eye, innervation of muscles of mastication, facial expression and tongue, and in transmitting information from chemoreceptors to the respiratory center [36], 37, and thus, defects in cranial nerve development may be relevant to reduced survival of *Jarid1b* knockouts. For example, the hypoglossal nerve (XII), which is dysmorphic in *Jarid1b* knockouts, innervates the muscles of the tongue, crucial for upper airway aperture during breathing.

Next, we analyzed *Jarid1b* expression during the time of mouse development when cranial nerves are specified. From embryonic day 8, the hindbrain becomes transiently partitioned along the anterior-posterior (AP) axis in a series of 8 rhombomeres that influence the spatial distribution of neuronal types [34]. Using the lacZ-Neo-reporter cassette present in the targeting construct [28], we observed high ubiquitous expression of *Jarid1b* in embryonic but not extraembryonic tissues at E8.5 (Figure S4). Moreover, in agreement with previous reports [38], at E12.5 and E14.5, *Jarid1b* expression was observed in several neural tissues including the fore- and hindbrain, neural retina, spinal cord and dorsal root ganglia as well as other tissues (Figures S5 and S6), indicating that *Jarid1b* could be involved in the development of several organs.

Cranial nerve development is imparted by genes involved in AP patterning and rhombomere specification, neuronal determination or survival and axonal migration [37]. Compartmentalization of the hindbrain, and in particular rhombomeres 3 and 4, have emerged as territories for the maintenance of breathing frequency after birth [34]. Rhombomeres are characterized by specific patterns of *Hox* gene, *Krox20* (*Egr2*) and *Kreisler* (*Mafb*) expression, leading us to analyze expression of these genes by RNA in situ hybridization in *Jarid1b* ^−/−^ embryos. However, we did not observe any defects in the hindbrain patterning of E8.75 embryos (Figure 3C), suggesting that other mechanisms are responsible for spinal nerve abnormalities in *Jarid1b* ^−/−^ embryos.

### Eye development is frequently disturbed in *Jarid1b* knockout embryos

**Figure 4:**
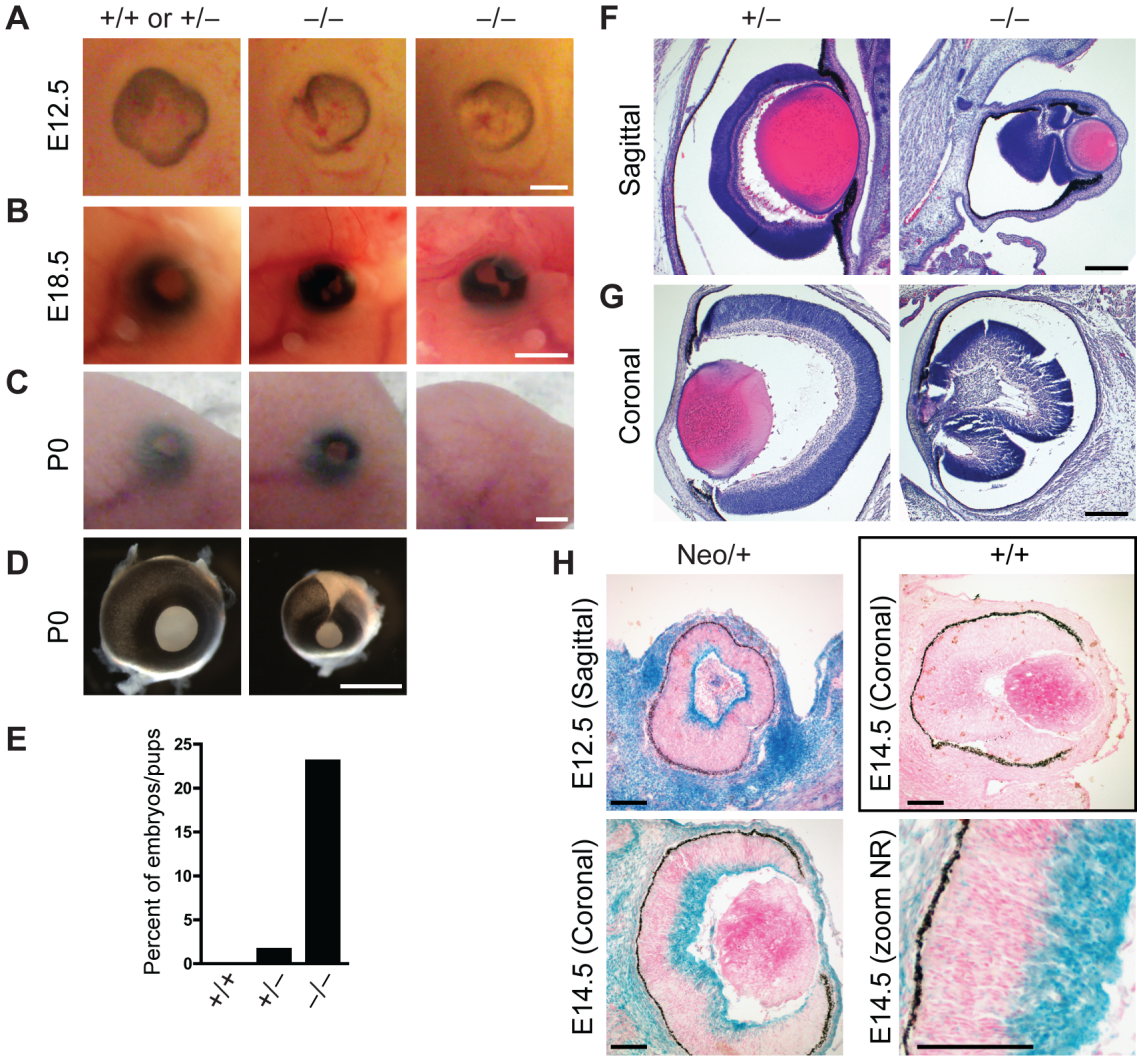
Defects in eye development in Jarid1b knockout embryos. (*A*) External morphology of E12.5 eyes. Ventral is to the left. Note that the optic fissure is not completely closed in the *Jarid1b* knockouts while it is closed in controls (pooled +/+ and +/−). Scale bar, 200 µm. (*B*) Eyes of E18.5 embryos. Scale bar, 1 mm. (*C*) Eyes of newborn mice. Note that one of the knockouts has an open eyelid (left) while the other knockout completely lacks the eye (right). Scale bar, 1 mm. (*D*) Isolated eyes of newborn mice. Scale bar, 1 mm. (*E*) Frequency of the externally visible defects described in (*B*) and (*C*) for wild-types (n = 47), heterozygotes (n = 123) and knockouts (n = 78). (*F*, *G*) Hematoxylin and eosin staining of paraffin-embedded embryos at E18.5 showing the left eye cut sagittally (*F*) and the right eye cut coronally (*G*). Note that both knockout eyes are microphthalmic and the neural retina is misfolded. Scale bar, 100 µm. (*H*) Staining for β-galactosidase on sections of E12.5 and E14.5 eyes representing *Jarid1b* expression. For E14.5, a zoom on the neural retina (NR) is shown at the bottom right. Staining of a wild-type embryo is shown as negative control.

In addition to sporadic cases of exencephaly, we frequently observed defects in eye development in *Jarid1b* ^−/−^ embryos and pups (Figure 4A–4D). In the most severe cases, eyes were completely absent (anophthalmia; Figure 4C). Other embryos exhibited microphthalmia (Figure 4B and 4D) or an incomplete closure of the optic fissure (Figure 4A and 4D). Moreover, after birth, the eyelid was often found open in *Jarid1b* ^−/−^ pups while it was closed in control mice at this time (Figure 4C). Altogether, externally visible eye defects were observed in approximately 22 percent of *Jarid1b* ^−/−^ embryos and pups (Figure 4E), but never in the *Jarid1b* knockouts that survive to adulthood. Histological analysis of two microphthalmic *Jarid1b* ^−/−^ eyes at E18.5 revealed a misfolding of the neural retina and a much smaller lense (Figure 4F and 4G). To test whether Jarid1b is expressed in the developing eye, we performed β-galactosidase stainings on sections of E12.5 and E14.5 eyes from targeted *Jarid1b* embryos (Figure 4H). At both stages, *Jarid1b* is specifically expressed in the inner layer of the neural retina, which contains retinal ganglion cells. Thus, *Jarid1b* seems required for the proper development of a mouse neurosensory organ, the eye.

### *Jarid1b* knockout embryos display homeotic transformations of the skeleton

We have previously shown that Jarid1b binds to the transcription start sites of many developmental regulators in mouse ESCs, many of which are also bound by Polycomb group proteins [28]. Therefore, we speculated that Jarid1b might also regulate Polycomb target genes *in vivo*. *Hox* genes represent classical Polycomb targets and their misexpression in Polycomb mouse mutants results in transformations of the axial skeleton [39], [40].

**Figure 5:**
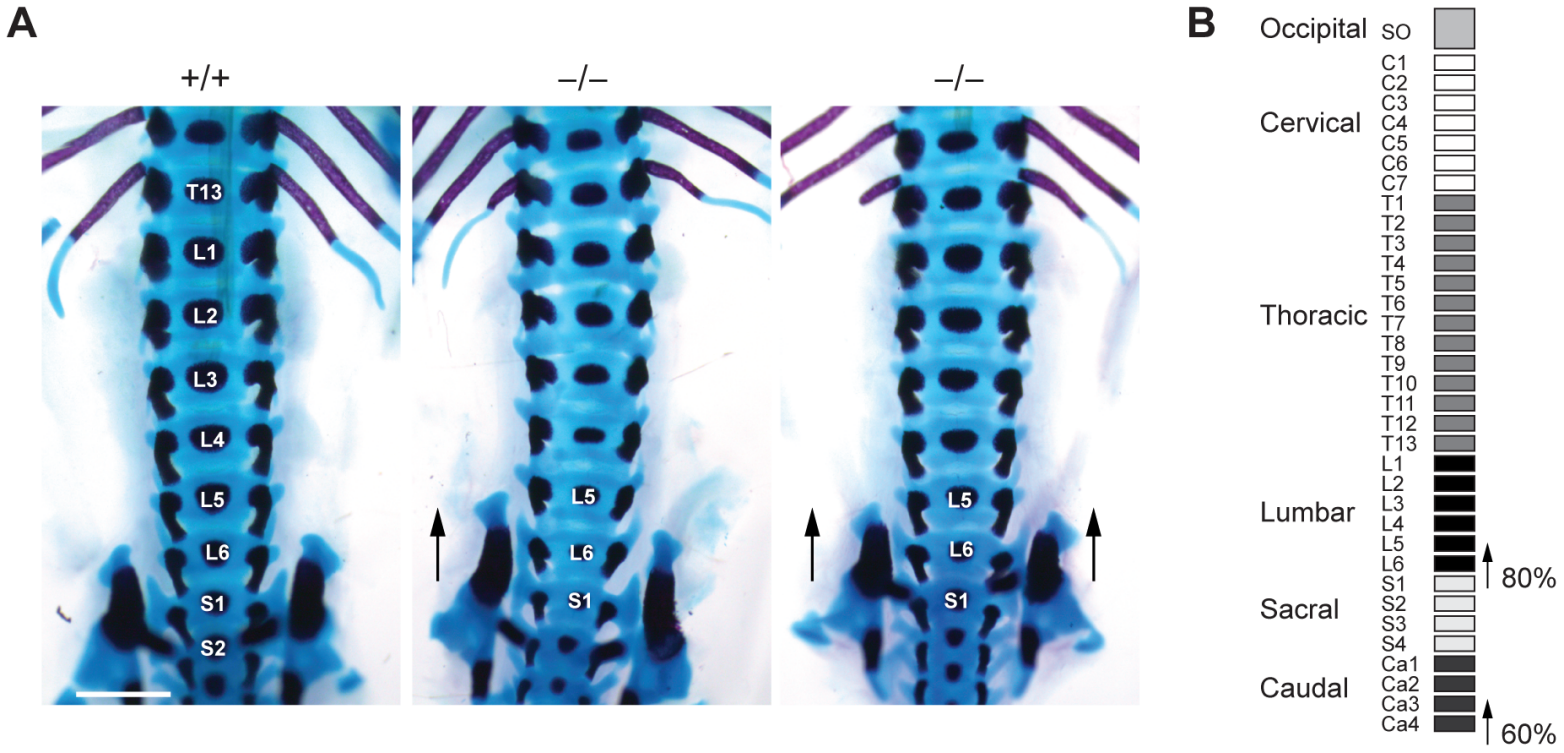
Skeletal transformations in Jarid1b knockout embryos. (*A*) Skeletal preparations of E17.5 *Jarid1b* embryos stained with Alcian blue (cartilage) and Alizarin red (bone). Shown is a ventral view of the lumbar-sacral region. Note that the sacroiliac joints are attached asymmetrically in the knockout shown on the left (n = 5/10) while the joints are symmetrically attached to L6 in the knockout shown on the right (n = 3/10). Scale bar, 1 mm. (*B*) Schematic representation of the frequency of posterior skeletal transformations in *Jarid1b* knockout embryos.

To investigate whether such transformations are also present in *Jarid1b* mutants, we stained skeletal preparations of E17.5 embryos to visualize cartilage and bone. While we did not observe any defects in the anterior region of the vertebral column (occipito-cervico-thoracic region), we found a transformation of the 26th vertebra, which is supposed to be the last lumbar vertebra (L6) into the first sacral vertebrae (S1) (Figure 5A and 5B). Moreover, we also observed a transformation of the 34th vertebra (Figure 5B and Figure S7). Thus, *Jarid1b* ^−/−^ embryos display posterior transformations of the skeleton, which similar to Polycomb mutants are not completely penetrant [39], [40].

### H3K4me3 is increased at normally repressed genes in early knockout embryos

To identify genes in addition to the *Hox* genes that might be misregulated in *Jarid1b* ^−/−^ embryos, we focused on an early embryonic stage (E8.5) where morphological defects were not yet observed. We expected that several of the phenotypes observed in the *Jarid1b* mutants arise from misspecification events early in development, as genes involved in eye specification, neural tube closure and hindbrain patterning start to be expressed from E8.0 [41], [42].

**Figure 6:**
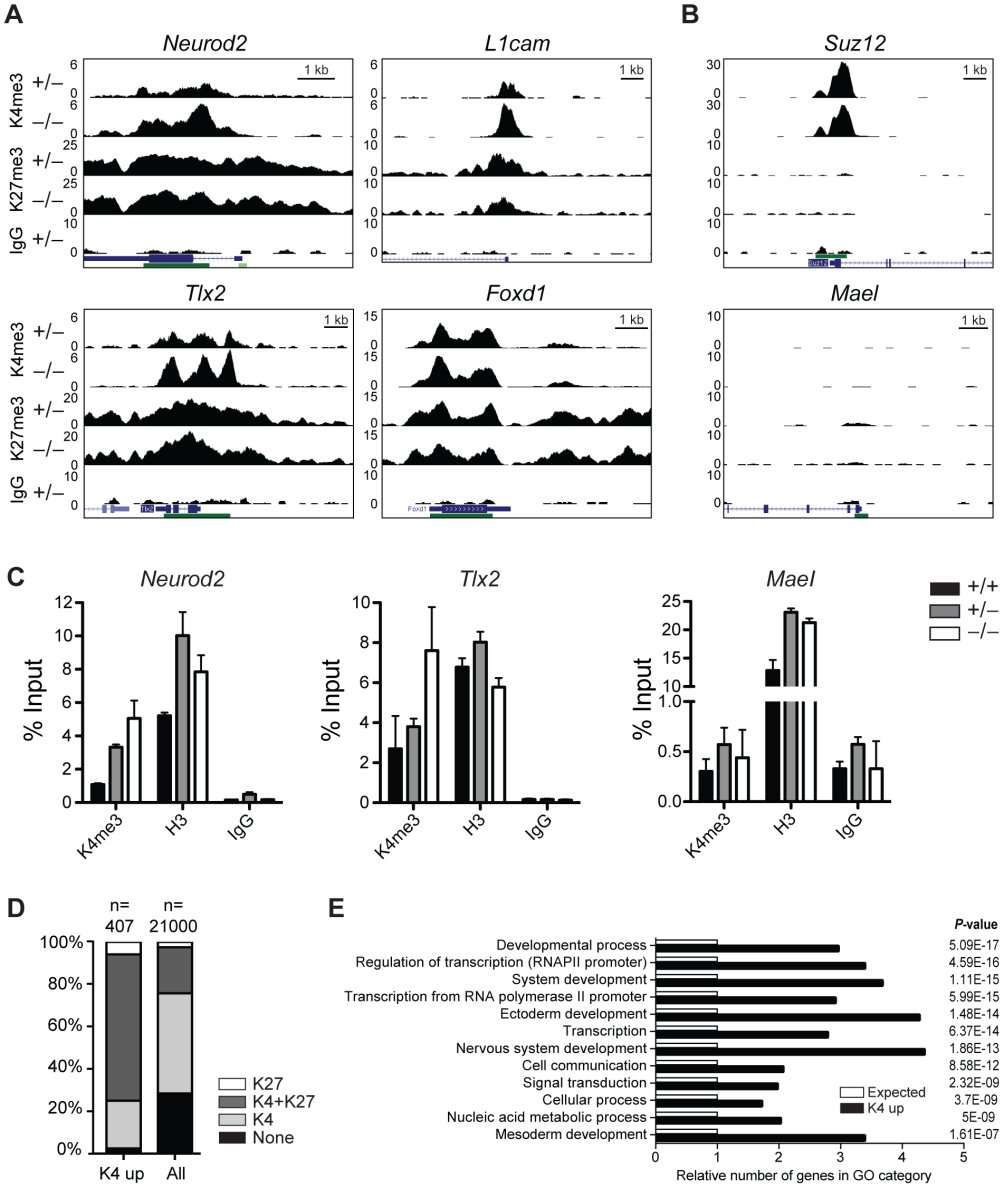
H3K4me3 is increased at bivalent and repressed genes in early *Jarid1b* embryos. (*A*, *B*) Genome wide profiles of H3K4me3 and H3K27me3 were determined by ChIP-seq in the head region of *Jarid1b* heterozygous and knockout embryos at E8.5. Representative loci with elevated (*A*) and unchanged (*B*) H3K4me3 levels in the knockout are shown. y-axis denotes number of sequence tag reads/million. Schematic presentation of Refseq transcripts is in dark blue, and dark green bars represent CpG islands. (*C*) ChIP-qPCR for H3K4me3, H3 and IgG in E8.5 embryos. Error bars represent S.D. of three PCR amplifications. (*D*) Percentage of H3K27me3, H3K4me3/H3K27me3, H3K4me3 and unmodified genes among genes with elevated H3K4me3 levels compared to all genes. (*E*) Gene ontology analysis of genes with elevated H3K4me3 levels generated using PANTHER. The 12 top scoring categories are shown.

First, we performed chromatin immunoprecipitation (ChIP) followed by sequencing (seq) of head regions of E8.5 embryos (Figure S8A) for H3K4me3 and H3K27me3 to identify genes that change their chromatin state and thus might become misregulated in *Jarid1b* ^−/−^ embryos. By this analysis, we identified 492 peaks with increased H3K4me3 levels in *Jarid1b* knockouts versus heterozygotes, whereas only 27 peaks were detected in the reverse comparison (Figure S8B). Representative examples of loci with increased H3K4me3 in the knockouts as well as loci with unchanged chromatin states are shown in Figure 6A and 6B, respectively. The results were validated in an independent experiment by ChIP-qPCR showing that the differences in H3K4me3 are reproducible (Figure 6C). Comparison of genes with increased H3K4me3 in knockout embryos with all genes revealed an enrichment of repressed (H3K27me3 positive) and bivalent (H3K4me3/H3K27me3 positive) genes among genes with increased H3K4me3 (Figure 6D and Figure S8C), suggesting that aberrant active histone marks accumulate mainly at genes that are usually not actively transcribed. Gene ontology analysis of genes with increased H3K4me3 in the *Jarid1b* ^−/−^ embryos identified regulators of transcription and development including genes involved in ectoderm, nervous system and skeletal development as significantly overrepresented (Figure 6E and Figure S8D). To identify genes that are directly bound and regulated by Jarid1b, we also attempted ChIP experiments for Jarid1b in E8.5 embryos but unfortunately the results were of low quality due to very limited amounts of starting material. Instead, we compared genes with elevated H3K4me3 in *Jarid1b* ^−/−^ embryos with genes bound by Jarid1b in ESCs [28] and found that approximately one quarter was bound by Jarid1b in ESCs (Figure S8E). Thus, it is likely that some of the genes with increased H3K4me3 are also Jarid1b targets during early mouse development.

Next, we performed gene expression analysis of mRNA isolated from E8.5 *Jarid1b* heterozygotes and knockouts. Except for *Jarid1b*, we did not identify any genes that were more than 2-fold changed in the knockouts (Figure S8F). We validated a number of genes by RT-qPCR and confirmed that *Jarid1b* was not expressed in the knockouts, whereas *Jarid1a* and *Jarid1c* as well as *L1cam* and *Pax2* remained unchanged (Figure S8G). Taken together, while we detected increased levels of H3K4me3 at a number of developmental regulators early in embryogenesis, these chromatin changes do not translate into detectable global transcriptional changes at this stage of development.

### Increased levels of H3K4me3 in *Jarid1b* knockout embryos during development

**Figure 7:**
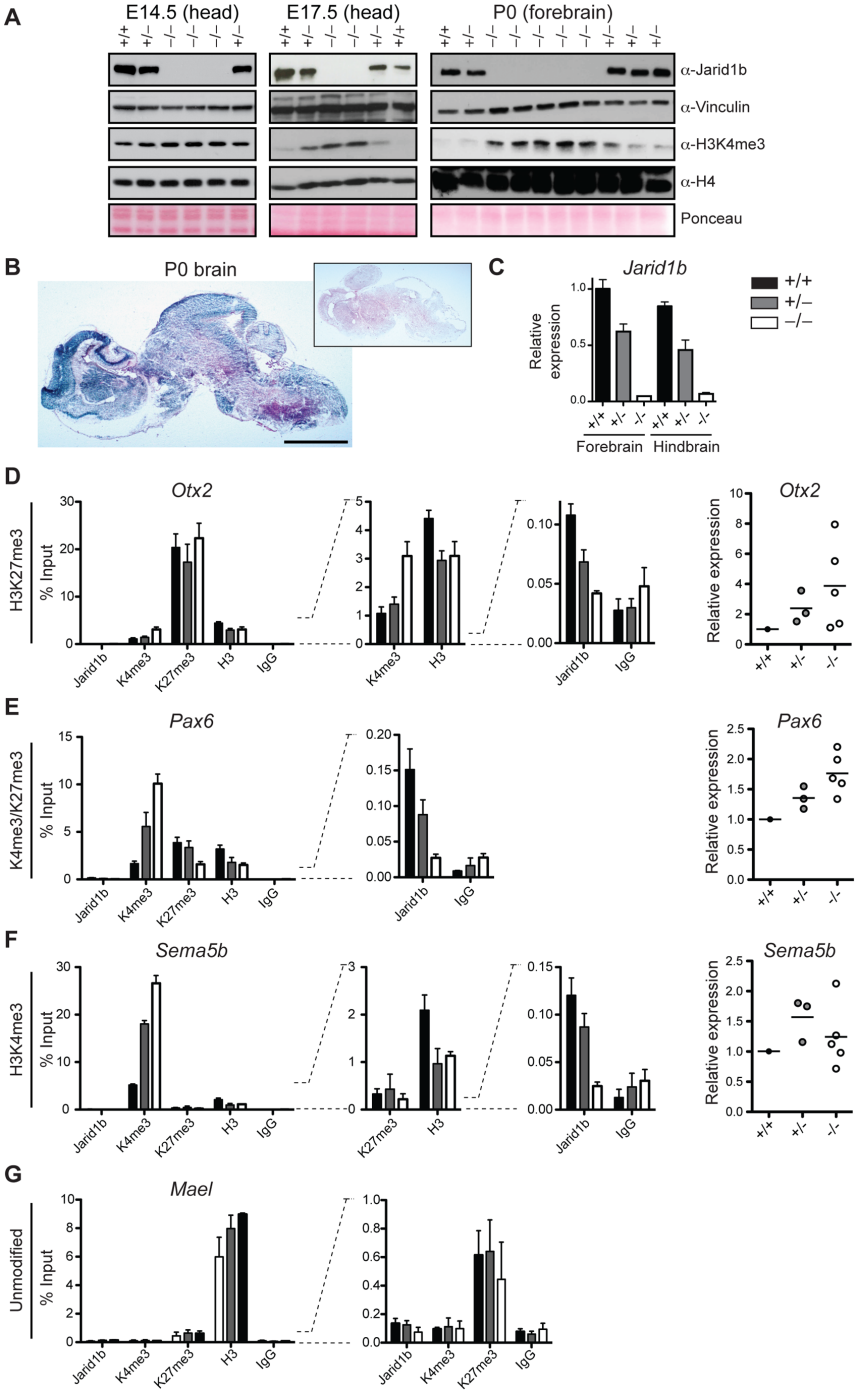
Loss of *Jarid1b* leads to increased global levels of H3K4me3 and higher expression of key developmental regulators during embryogenesis. (*A*) Immunoblots for Jarid1b and H3K4me3 of *Jarid1b* embryos at different developmental stages. Vinculin, H4 and Ponceau serve as loading controls. (*B*) Staining for β-galactosidase on sections of P0 brains representing *Jarid1b* expression. Staining of a wild-type brain is shown as negative control (boxed inset). Scale bar, 2 mm. (*C*) Expression of *Jarid1b* in fore- and hindbrains of wild-type, heterozygous and knockout newborns (normalized to *β-actin*). (*D*–*G*) Left: ChIP-qPCR for Jarid1b, H3K4me3, H3K27me3, H3 and IgG in forebrains of P0 pups. Error bars represent S.D. of three PCR amplifications. Right: Expression of indicated genes in forebrains analyzed by RT-qPCR (normalized to *β-actin*). Each dot represents an individual embryo. *MaeI* expression was below detection. One representative gene is shown for each chromatin state: (*D*) H3K27me3, (*E*) H3K4me3/H3K27me3, (*F*) H3K4me3 and (*G*) unmodified.

Deletion of *Jarid1b* in ESCs leads to a global increase in H3K4me3, while global H3K4me3 levels remain unchanged in *Jarid1b* depleted neural stem cells isolated from E12.5 embryos [28]. Likewise, depletion of *JARID1B* in MCF7 cells [43] or depletion of *Jarid1a* in mouse embryonic fibroblasts [24] did not result in a global elevation of H3K4me3. To analyze the effect of *Jarid1b* depletion *in vivo*, we prepared protein extracts from different stages of embryos. We confirmed lack of Jarid1b protein in all knockout embryos analyzed (Figure 7A). While we detected little change in H3K4me3 by immunoblotting in heads of E12.5 (data not shown) and E14.5 *Jarid1b* ^−/−^ embryos, global H3K4me3 levels were strongly increased in heads of late (E17.5) *Jarid1b* ^−/−^ embryos and in forebrains of *Jarid1b* ^−/−^ newborns (Figure 7A). These results suggest that H3K4me3 accumulates in *Jarid1b* knockouts as embryonic development proceeds, while H3K4me3 levels remain fairly constant during normal fetal development (Figure S9).

### Increased H3K4me3 at transcription start sites in brains of newborn *Jarid1b* knockouts

Next, we wanted to know at which classes of genes H3K4me3 accumulates in brains of newborn mice. Since the brain is a complex and heterogeneous organ, we first determined whether *Jarid1b* expression is limited to specific regions at this stage of brain development. However, β-galactosidase stainings on sections of brains from newborns revealed high overall expression of *Jarid1b* (Figure 7B). In addition, RT-qPCR analysis showed similar expression of *Jarid1b* in fore- and hindbrain, which is reduced in heterozygotes and lost in knockouts (Figure 7C). Thus, we divided the brain into forebrain and hindbrain for ChIP experiments and selected a number of genes that represent different chromatin states (Figure 7D–7G and S10). We observed increased H3K4me3 at repressed (H3K27me3-positive) genes, including *Otx2*, *Pax9*, *HoxB5* and *Hesx1*, and at active (H3K4me3-positive) genes (*Sema5b*), but not at unmodified genes in P0 forebrains. Some bivalent genes, for example *Pax6*, showed increased H3K4me3 and slightly reduced H3K27me3, while others remained unchanged (e.g. *Neurod2*). Similar results were obtained in independent ChIP experiments using forebrain or hindbrain. These data suggest that H3K4me3 is increased at transcription start sites in late stages of brain development.

To test which genes are directly bound by Jarid1b, we also performed ChIP for Jarid1b (Figure 7D–7G and Figure S10). We detected Jarid1b binding at transcription start sites of bivalent (*Pax6*) and H3K4me3-positive (*Sema5b*) genes, which is in agreement with our previous findings in ESCs [28]. Moreover, we detected low levels of Jarid1b binding (2- to 4-fold above background) at several of the H3K27me3-positive loci with increased H3K4me3 in the *Jarid1b* knockouts (*Otx2*, *Pax9*, *Hoxb5*), suggesting that elevated levels of H3K4me3 at many of these loci are due to a loss of direct association of Jarid1b.

### Increased expression of several key transcription factors in *Jarid1b* knockouts

To determine whether changes in chromatin modifications are accompanied by differences in expression, we performed RT-qPCR in P0 brains of controls and *Jarid1b* knockouts (Figure 7D–7F and Figure S10). We detected increased levels of the transcription factor *Otx2* in forebrains of *Jarid1b* ^−/−^ newborns. Furthermore, in line with a shifted balance of H3K4me3 versus H3K27me3, expression of the neural master regulator *Pax6* was increased in *Jarid1b* ^−/−^ P0 brains. In contrast, expression of actively transcribed genes, like *Sema5b*, was unchanged despite higher levels of H3K4me3. We conclude that *Jarid1b* mutants accumulate higher levels of H3K4me3 and show increased expression of genes important for regulating embryonic development.

Next, we analyzed at which stage between E8.5 and P0 changes in gene expression arise in *Jarid1b* knockouts. While we did not detect transcriptional changes at E8.5, expression of *Otx2*, *Pax6* and *Sema5b* was increased in heads of E12.5 *Jarid1b* knockout embryos compared to controls (Figure S11A). Since the transcription factor Pax6 controls the balance between neural stem cell (NSC) self-renewal and neurogenesis [44], we tested whether deletion of Jarid1b affected this balance. Sorting of NSCs and neuronal progenitor cells (NPs) from E12.5 brains (Figure S11B) revealed a slight (but not significant) increase in NSCs in *Jarid1b* knockouts and no change in NPs. Similarly, global levels of neuron and astrocyte markers remained unchanged in P0 brains (Figure S9B), which is in agreement with normal gross morphology of Jarid1b knockout brains (Figure S3F). Thus, the detectable changes in gene expression observed in Jarid1b knockout mice does not appear to be a result of abnormal numbers of NSCs or NPs.

Finally, we tested whether increased expression of *Otx2* and *Pax6* correlated with survival. However, as shown in Figure S11C, we did not detect a significant difference in expression of *Otx2* and *Pax6* in brains of newborns that were alive 2 hours after caesarean delivery versus newborns that died immediately. In contrast, the expression of *Otx2* was significantly higher in the adult brain of surviving knockout animals as compared to wild type (Figure S11D), suggesting that transcriptional regulation by Jarid1b is not restricted to embryogenesis only, but affects selected genes rather than global transcription.

## Discussion

Embryonic development is regulated by transcription factors as well as chromatin-mediated processes resulting in tissue-specific gene expression. Here, we show that the histone demethylase Jarid1b is required for faithful mouse embryonic development (see model in Figure S12). Deletion of *Jarid1b* results in major neonatal lethality caused by an inability of the newborn mice to establish breathing. *Jarid1b* mutant embryos display a number of defects related to neural systems, including the misorganization of cranial and spinal nerves as well as increased incidence of exencephaly that might contribute to neonatal lethality. Respiratory rhythmogenic circuits in the brainstem of *Jarid1b* mutant embryos appear intact since a spontaneous motor output on cervical nerves was observed under *in vitro* conditions. In agreement, *Krox20* and *Kreisler*, essential genes involved in specification of respiratory-related rhombomeres, are also not affected in mutant embryos. Thus, we speculate that the breathing problems of *Jarid1b* mutant neonates may stem from either compromised pattern forming circuits controlling airway patency, or an inability of the rhythmic motor output to reach respiratory muscles, caused by defects in cranial and spinal nerve development. Several other organs important after birth appeared undisturbed. However, the spectrum of physiological systems required for neonatal survival is large [30] and we cannot exclude that there are other subtle defects that manifest in secondary physiological problems interfering with survival of *Jarid1b* knockouts.

Previous *in vitro* studies of ESCs with either reduced [28] or increased [27] levels of Jarid1b have reported a role for Jarid1b during differentiation of ESCs into neurons. This raises the question of why Jarid1b is specifically required in neural systems. During embryogenesis, Jarid1b is expressed in several neural organs including the brain, spinal cord and eye, but also in a number of other systems (this study, [38]). Moreover, in ESCs, Jarid1b is targeted to transcription start sites of genes that regulate development, including genes involved in neurogenesis and ectoderm development [28]. Thus, a combination of tissue-specific expression and target gene selectivity might explain neural-specific phenotypes. It should be noted, however, that other systems are also affected by Jarid1b depletion, exemplified by homeotic transformation of the skeleton (this study) or slightly reduced expression of meso- and endodermal markers during embryoid body differentiation of ESCs [28]. Interestingly, knockdown of *Jarid1b* in the retina of newborn mice leads to abnormal morphology of rod photoreceptor cells and misregulation of rod-expressed genes [45], supporting a role for Jarid1b in neuronal cells of the eye. In addition, other Jarid1 family members have reported functions in behavior and/or neurulation, suggesting that these processes are susceptible to changes in H3K4 methylation. *Jarid1a* knockout mice display abnormal clasping of the hindlimbs [24], while mutations of human JARID1C occur in patients with X-linked mental retardation [46]. Knockout of *Jarid1c* in the mouse results in embryonic lethality due to defects in neurulation and cardiogenesis [47]. Taken together, while several Jarid1 family members are involved in the control of neural systems, they may regulate different aspects of development and cannot fully compensate for the absence of other Jarid1 members resulting in gene-specific phenotypes.

To determine how Jarid1b contributes to the regulation of mouse development, we analyzed global as well as gene-specific histone methylation levels in *Jarid1b* ^−/−^ embryos. Consistent with the previously reported catalytic activity of Jarid1b [23], [43], H3K4me3 was increased around transcription start sites of developmental regulators already early during mouse development, particularly at genes that are normally in a repressed or poised state. At this early stage of development, we could not detect any global changes in gene expression levels, but we cannot exclude that expression of some genes might be affected in a subset of cells as the E8.5 mouse embryo is composed of many distinct cell layers. For example, the development of the eye initiates at E8.0 with the evagination of the optic pit from a subset of cells in the diencephalon [41]. In analogy, increased H3K4me3 at transcription start sites may reflect small increases in many cells of the early embryo or result from large increases in a subset of cells. Increased H3K4me3 around transcription start sites may render the associated genes more susceptible to later activation, especially during developmental time windows or in specific cell types where additional signaling molecules create a competent transcriptional state.

As embryonic development proceeds, a global increase in H3K4me3 becomes detectable. Locally, similar to early embryos, increased H3K4me3 is present at the transcription start sites of genes in *Jarid1b* ^−/−^ brains. Even though altered H3K4me3 *per se* may not be sufficient to induce transcriptional changes or cell fate conversions [48], it may have functional consequences when prompt responses to signaling events are required or for the fine control of steady state transcript levels [49]. Indeed, we detected increased expression of *Pax6* and *Otx2*, two master regulators of eye and neural development (reviewed in [50], [51]), in forebrains of *Jarid1b* ^−/−^ pups. For both *Pax6* and *Otx2*, it was shown that not only deletion but also overexpression affect eye and neural lineage development [44], [52], [53].

Binding of Jarid1b itself was found at H3K4me3-positive genes (both active and bivalent), which is in agreement with previous ChIP-seq data [28], [54]. Interestingly, the overlap between Jarid1b and Polycomb target genes was functionally supported in this study by the observation that *Jarid1b* knockout embryos show homeotic transformations of the skeleton, which is a hallmark of Polycomb mutant mice. Moreover, the observation that Jarid1b is bound to several repressed genes that are marked by aberrant H3K4me3 in the knockouts, suggests that Jarid1b is directly required to prevent aberrant accumulation of active chromatin modifications at developmental regulators during embryogenesis.

Most of the phenotypes that we observed in *Jarid1b* knockouts occurred with incomplete penetrance. This is not uncommon and has been reported for other histone demethylases [55] but also transcription factors [56]. The penetrance is often affected by the genetic background of the mice [50], [55]. This might also explain the difference in survival of our knockout mice compared to a previous study [25]. To test this hypothesis, we crossed our *Jarid1b* mutant mice that were derived on a C57BL/6 background into a mixed C57BL/6/129 genetic background (Figure S13). On the mixed background, 40% of knockouts die after birth, compared to 70% on a C57BL/6 background. Besides, we observed a similar frequency of exencephaly and a reduced response of the newborns to pinching stimuli, while no eye phenotypes were detected on the mixed background. These results suggest that different genetic background of mice strains used, could partly explain the divergence of obtained results.

Moreover, many disease-causing mutations only have detrimental defects in a subset of individuals, and phenotypic discordance remains even in the absence of genetic and environmental variation. It was shown that feedback induction of genes with related functions differs across individuals leading to a buffering of stochastic developmental failure through redundancy [57]. In the *Jarid1b* mutants, we did not observe upregulation of other Jarid1 members at the transcript level. However, we cannot exclude that protein levels are increased, or that these proteins are preferentially recruited to Jarid1b target sites to compensate for lack of Jarid1b. In addition, systematic analysis of transcript levels and their correlation with phenotypes has shown that variability in gene expression underlies incomplete penetrance [58]. It was proposed that fluctuations in gene expression can be controlled by wild-type developmental networks. In contrast to transcription factors that can induce cell fate conversions, chromatin modification are thought to rather fine tune transcription. In *Jarid1b* mutant embryos, both repressed and bivalent genes acquire increased levels of H3K4me3, which might render these genes more susceptible to unscheduled activation. Indeed, we observed increased expression of *Pax6* and *Otx2* in newborn knockout brains. Raj et al. [58] propose a model in which expression must surpass a threshold during a window of development. The same might be true for histone modifications. In plants, progressive increase in H3K27me3 was shown to be capable of switching a bistable epigenetic state of an individual locus [59]. While global levels of H3K4me3 are unchanged during early embryogenesis, H3K4me3 accumulates in the *Jarid1b* knockouts as embryonic development proceeds. For some genes or in specific cell types, this might lead to a switch in the balance of active versus repressive histone modifications, and if this coincides with a developmental window of transcriptional potency, it might affect phenotypic outcomes. Moreover, since Jarid1b binds to a large number of target genes [28], it could be expected that a wide range of phenotypes with varying severity is observed in *Jarid1b* knockout embryos.

The functions of histone demethylases *in vivo* are starting to emerge, however, in many cases the mechanisms of their action remain to be elucidated. Here, we present a detailed analysis of the role of the H3K4me2/3 demethylase Jarid1b during mouse development. In the adult organism, Jarid1b expression is also observed but it becomes more restricted. Since high expression of Jarid1b is detected during meiosis and in adult testis [29] as well as in several types of cancer [26], Jarid1b has been proposed to belong to the family of testis-cancer antigens [60]. In future studies, it will be very interesting to characterize the function of Jarid1b in adult mice as Jarid1b presents a potential drug target for anti-cancer therapies and an understanding of its *in vivo* role will help to guide targeting efforts.

## Materials and Methods

### Animals

The derivation of targeted (*Jarid1b* ^Neo/+^) and conditional (*Jarid1b* ^F/F^) Jarid1b mice has been previously described [28]. Conditional *Jarid1b* mice were crossed with *Cmv*-cre transgenic mice [61] to obtain heterozygous mice, which were further inter-crossed to generate *Jarid1b* knockouts. *Jarid1b* mice were maintained on a C57BL/6 background, unless otherwise stated. All mouse work was approved by the Danish Animal Ethical Committee (“Dyreforsøgstilsynet”).

For cesarean deliveries, timed matings were setup using *Jarid1b* ^+/−^ mice to generate experimental pups. *Jarid1b* ^+/+^ mice were used for foster mothers. Pregnant *Jarid1b* ^+/−^ females were injected subcutaneously with 100 µl of Promon (50 mg/ml, Boheringer Ingelheim) at E16.5 and E18.5 to prevent natural birth. Pups were delivered at E19.5 by caesarean, massaged gently to stimulate breathing and placed on a 37°C warm plate during initial examination. Pups were then placed with a foster mother and examined regularly during the first 24 hours after delivery. Dexamethasone (Sigma) or saline control was administered subcutaneous (0.4 mg/kg) to pregnant females at E17.5 and E18.5 [33].

### Histology

Histological analysis was performed according to standard procedures. Briefly, embryos or tissues were fixed over night in 4% buffered formaldehyde and subsequently incubated in baths of buffered formaldehyde, 96% ethanol, 99% ethanol, xylene and paraffin. Paraffin blocks were cut into 8–10 µm sections on a microtome (Microm HM355S). Sections were deparaffinised, stained with hematoxylin and eosin, dehydrated and mounted using VectaMount (Vector).

### Nerve recordings in brainstem–spinal cord preparations

The neuraxis in caesarean delivered E18.5 embryos was removed by dissection in an ice cold, oxygenated (95% O_2_, 5% CO_2_) solution containing 250 mM glycerol, 3 mM KCl, 5 mM KH_2_PO_4_, 36 mM NaHCO_3_, 10 mM D-(+)-glucose, 2 mM MgSO_4_ and 0.7 mM CaCl_2_. Brainstem-spinal cord preparations, which contained the entire brainstem and the cervical part of the spinal cord, were placed in a 2 ml recording chamber with a temperature of 29°C and was constantly superfused at a rate of 2 ml/min with preheated oxygenated (95% O_2_, 5% CO_2_) artificial cerebrospinal fluid solution (ACSF). The ACSF solution contained 130 mM NaCl, 5.4 mM KCl, 0.8 mM KH_2_PO_4_, 26 mM NaHCO_3_, 30 mM D-(+)-glucose, 1 mM MgCl_2_ and 0.8 mM CaCl_2_. Glass-pipette suction electrodes (tip-diameter of 40–160 µm, A-M Systems, Carlsborg, USA) were placed on C3, C4, or C5 rootlets to record spontaneous respiratory-related nerve-activity. Nerve potentials were amplified by a custom-built nerve amplifier (×50,000), filtered at DC-2 KHz, and digitized (2.5 KHz) by a PCI–6289, M Series A/D-board (National Instruments, Austin, USA) controlled by Igor Pro (Wavemetrics, Lake Oswego, USA) software.

### Beta-galactosidase stainings

For whole-mount beta-galactosidase stainings, embryos were fixed in PBS with 0.25% glutaraldehyde for 10–30 min, washed in PBS and stained with PBS containing 0.02% NP40/IGEPAL, 0.01% sodium deoxycholic acid, 2 mM MgCl_2_, 20 mM Tris-HCl pH 7.4, 5 mM Potassium ferrocyanide and 5 mM Potassium ferricyanide until desired colour intensity. After post-fixation in 4% paraformaldehyde over night at 4°C, embryos were passed through a glycerol gradient incubating several days at each concentration, and finally stored in 100% glycerol.

For beta-galactosidase stainings of cryo-sections, embryos were fixed in 0.2% paraformaldehyde over night at 4°C, incubated in PBS with 2 mM MgCl_2_ and 30% sucrose over night at 4°C, embedded in OCT (TissueTek, Sakura) and stored at −80°C. Samples were cut into 8 µm sections on a cryostat (Leica CM3050), post-fixed in 0.2% paraformaldehyde for 10 min on ice, washed in PBS with 2 mM MgCl_2_, incubated in PBS with 2 mM MgCl_2_, 0.01% deoxycholic acid and 0.02% NP40 for 20 min on ice, and stained as described above. Sections were washed, counter-stained with eosin, passed through an ethanol gradient, incubated in xylene and mounted in VectaMount (Vector).

### In situ hybridisation

Whole-mount in situ hybridization of mouse embryos was performed as previously described [62].

### Neurofilament staining

Embryos were isolated at E10.5, fixed in 4% paraformaldehyde for 2 hours and stored in 100% methanol at −20°C. After bleaching with methanol/H_2_O_2_, embryos were rehydrated, blocked in PBS with 2% milk and 0.1% triton (PBSMT) and stained over night at 4°C with anti-neurofilament antibody (Developmental Studies Hybridoma Bank, 2H3, 1∶50). Embryos were washed in PBSMT, and incubated over night at 4°C with peroxidase-conjugated goat anti-mouse IgG (Jackson ImmunoResearch Laboratories, 111-035-146, 1∶500). After several washes in PBSMT, embryos were incubated in 0.3 mg/ml DAB (Sigma) in 0.5% NiCl2 for 30 min, H_2_O_2_ was added to a concentration of 0.0003% and the embryos incubated until the desired colour intensity was obtained. Finally, embryos were dehydrated through a methanol gradient and cleared in 1∶2 benzyl alcohol∶benzyl benzoate in glass containers.

### Skeletal preparations

For skeletal preparation, embryos were isolated at E17.5, eviscerated and the skin removed. Embryos were fixed over night in 100% ethanol, rinsed in 95% ethanol and stained over night in 0.15 mg/ml Alcian Blue in 95% ethanol containing 20% glacial acidic acid. Embryos were washed in 95% ethanol, cleared in 1% KOH for 4 hours and stained with 50 mg/l Alizarin Red in 1% KOH over night at 4°C. Final clearing of embryos was performed in 1% KOH for 3 hours and through a gradient of 1% KOH/glycerol until final storage in 100% glycerol.

### Antibodies

Antibodies used in this study include anti-Jarid1b (DAIN) [28], anti-H3K4me3 (Cell Signaling, C42D8), anti-H3K27me3 (Cell Signaling, D18C8), anti-H3 (Abcam, 1791), anti-H4 (Millipore, 05-858), anti-ß-III-tubulin (Sigma, T8660), anti-Glial Fibrillary Acidic Protein (GFAP) (DakoCytomation, Z0334), anti-Vinculin (Sigma, V9131) and anti-ß-tubulin (Santa Cruz, sc-9104).

### Flow cytometry

Whole brain from E12.5 was dissociated, filtered, resuspended in PBS with 5% FBS and stained for 20 min on ice using the following antibodies: anti-Prominin-1-biotin (MACS Miltenyi Biotec, 130-092-441), anti-CD-15-FITC (BD Biosciences 332778), anti-A2B5-APC (MACS Miltenyi Biotec, 130-093-582) and anti-CD24 (BD Biosciences, 553262) [63]. Cells were washed, incubated with secondary fluorescent-conjugated antibodies for 20 min on ice, washed again and resuspended in buffer for viability dye staining containing 7AAD (BioLegend 420404). Cells were analyzed on a FACS Aria flow cytometer (BD Biosciences). Single viable cells were gated into the following populations: Neural progenitors: CD15 low, Prominin low, CD24 high; Neural stem cells: CD15 high, Prominin high, CD24 low.

### Chromatin immunoprecipitation

Chromatin immunoprecipitation (ChIP) and ChIP-sequencing were performed as previously described [28]. MACS2 [64] was used to identify regions with increased histone methylation. For E8.5 embryos, five heads of embryos with 3–8 somites were pooled per ChIP, corresponding to approximately 100,000 cells in total. For ChIP and expression analysis of P0 brains, single brains were divided into fore- and hindbrain.

Base-calling and demultiplexing of raw sequencing data was performed using the standard Illumina pipeline (CASAVA, version 1.8.2) followed by alignment to the mouse genome (mm9 assembly) with bowtie (version 0.12.7) [65] using the following parameters: -S -m 1 mm9. Samtools (version 0.1.18) [66] and bedtools (version 0.1.18) [67] were applied for conversion of files between alignment formats. Furthermore, reads were extended to a total length of 250 bp (estimated DNA fragement size) in the 3′ direction. Various command-line utilities from UCSC (http://hgdownload.cse.ucsc.edu/admin/exe/linux.x86_64/) were used to generate normalized bigwig track files for viewing in the UCSC genome browser. The files were normalized to tags per million after removing duplicate reads. Peak calling was performed in MACS2 [64] using the following settings: –broad -f BAM -g mm.

### Gene expression analysis

Gene expression analysis was previously described [28]. For RNA isolation of E8.5 embryos, the RNAeasy Microkit (Qiagen) was used. For microarray analysis, four heads of E8.5 embryos were pooled per sample and three biological replicates analyzed. RNA was hybridized on mouse Gene 1.0 ST arrays by the RH Microarray Center at Rigshospitalet, Copenhagen, following Affymetrix procedures. Microarray data was analyzed using Gene Array Analyzer software [68] with default settings, P-value<0.05 and a log2 fold change of +/−1. The ChIP-seq and microarray data have been submitted to the Gene Expression Omnibus (GEO) database (GSE41174). Primer sequences are provided in Table S1.

## Supporting Information

Figure S1 Expression of Jarid1b is lost in knockout embryos. (A) Immunoblots of E12.5 *Jarid1b* embryos divided into head and body probed for Jarid1b and Vinculin (loading control). (B) Expression of *Jarid1b* in the head of wild-type (+/+), knockout (−/−) and heterozygous (+/−) embryos (E12.5) determined by quantitative RT-PCR (normalized to *β-actin* levels). Primers were designed to cover exons encoding the following functional domains: JmjN (E1–2), ARID (E3 and E3–4), JmjC (E11–12 and E13–14) and PHD2 (E23–24). (C) Expression of *Jarid1a*, *Jarid1c* (located on the *x* chromosome) and *Jarid1d* (located on the *y* chromosome) in heads of male E12.5 embryos.
(TIF)

Figure S2 Lung maturation in Jarid1b knockouts. (A) Expression of surfactant proteins in lungs of E18.5 *Jarid1b* knockout embryos analyzed by RT-qPCR. Each dot represents an individual embryo. (B) Survival curve of *Jarid1b* wild-type, heterozygous and knockout pups treated with saline (PBS) or dexamethasone prior to cesarean delivery to induce lung maturation. Note that the curves for wild-type PBS and wild-type dexamethasone treatment are identical.
(TIF)

Figure S3 Other organs required for neonatal survival are grossly normal in Jarid1b knockouts. (A) Skeletal preparations of E17.5 *Jarid1b* embryos stained with Alcian blue (cartilage) and Alizarin red (bone). Shown is a lateral view of the rib cage. Scale bar, 2 mm. (B) Hematoxylin and eosin staining of paraffin-embedded diaphragms of E18.5 embryos cut sagittally. Scale bar, 50 µm. (C) Preparation of the skull of E17.5 embryos (top: side view; bottom: view from below). Scale bar, 2 mm. (D) Hematoxylin and eosin staining of coronal sections of the palatal region of E18.5 embryos. Scale bar, 0.5 mm. (E) Dissection of E18.5 embryos visualizing the cardiovascular system. Scale bar, 1 mm. (F) Brains of newborn mice (P0). Scale bar, 2 mm. (G) Weight of *Jarid1b* heterozygote and knockout brains at P0 in mg (left) or as percent body weight (right). (H) Spontaneous C3 nerve-activity in brainstem-spinal cord preparations from E18.5 embryos. Cycle-triggered averages of the inspiratory bursts are shown to the right. Below: Frequency and duration of the spontaneous nerve-activity in pooled wild-type/heterozygous (n = 5) and knockout (n = 6) *Jarid1b* embryos. (I) Percent of newborn Jarid1b mice that reacted to a tail pinch stimulus with a strong (2), weak (1) or no response (0).
(TIF)

Figure S4 Jarid1b is widely expressed during embryogenesis. Staining for β-galactosidase in *Jarid1b* Neo/+ E8.5 embryos (A: embryo embedded in extraembryonic tissue; B: embryo dissected free of extraembryonic tissue; Scale bars, 0.5 mm) and E12.5 embryos (C; Scale bars, 1 mm) representing *Jarid1b* expression. Staining of *Jarid1b* +/+ embryos served as negative control.
(TIF)

Figure S5 Jarid1b expression in E12.5 embryos. (A) Staining for β-galactosidase on sagittaly cut 8 µm cryo-sections of E12.5 *Jarid1b* Neo/+ embryos representing *Jarid1b* expression. Scale bars, 100 µm. (B) Staining of *Jarid1b* +/+ E12.5 embryos (negative control). Scale bar, 1 mm. Top: anterior; left: dorsal.
(TIF)

Figure S6 Jarid1b expression in E14.5 embryos. (A) Staining for β-galactosidase on sagittal sections of E14.5 *Jarid1b* Neo/+ embryos. Scale bars, 250 µm. (B) Staining of *Jarid1b* +/+ E14.5 embryos (negative control). Scale bar, 1 mm. Top: anterior; left: dorsal.
(TIF)

Figure S7 Skeletal transformations in Jarid1b knockout embryos. Skeletal preparations of E17.5 *Jarid1b* embryos stained with Alcian blue (cartilage) and Alizarin red (bone). Shown is a ventral view of the sacral-caudal region. Note that the 34th vertebra (Ca4 in wild-type) lacks transverse processes in *Jarid1b* knockouts. Arrows indicate transverse processes in wild-type. S: sacral; Ca: caudal.
(TIF)

Figure S8 Characterization of loci with increased H3K4me3 in early Jarid1b knockout embryos. (A) Head region (boxed) of E8.5 embryos (3–8 somites) was used for ChIP. Scale bar, 0.2 mm. (B) Summary of ChIP-seq comparison of H3K4me3 and H3K27me3 between *Jarid1b* heterozygous and knockout embryos (E8.5). The number of peaks with increased histone methylation is shown as well as the fraction of peaks that overlaps with genes or CpG islands (CGI). (C) Number of genes with H3K27me3, H3K4me3/H3K27me3, H3K4me3 and unmodified genes among genes with elevated H3K4me3 levels (K4 up) compared to all genes. (D) Gene ontology analysis of genes with elevated H3K4me3 levels using the BP-FAT category in the DAVID software. (E) Venn diagram showing the overlap between genes bound by Jarid1b in ESCs and genes showing elevated levels of H3K4me3 in E8.5 *Jarid1b* knockout embryos. (F) Scatter blot of global gene expression microarrays comparing *Jarid1b* heterozygous and knockout E8.5 embryos (head regions). Red dot represents *Jarid1b*, the only gene that changes more than 1.5-fold. The correlation coefficient R^2^ is indicated. Values represent averages of three biological replicates. (G) Expression of *Jarid1b*, *Jarid1a*, *Jarid1c*, *L1cam* and *Pax2* in E8.5 embryos was determined by RT-qPCR (normalized to *β-actin*). Each dot represents an individual embryo.
(TIF)

Figure S9 H3K4me3 during brain development. (A) Immunoblots for H3K4me3 and Jarid1b from heads of wild-type E14.5 and E17.5 embryos and wild-type P0 brains. (B) Immunoblots for markers of neurons (β-III-tubulin) and astrocytes (Gfap) of *Jarid1b* wild-type, heterozygous and knockout P0 brains. Tubulin and Ponceau serve as loading controls.
(TIF)

Figure S10 H3K4me3 levels at genes and gene expression in Jarid1b knockout brains after birth. (A–C) Left: ChIP-qPCR for Jarid1b, H3K4me3, H3K27me3, H3 and IgG in forebrains of P0 pups. Error bars represent S.D. of three PCR amplifications. Right: Expression of indicated genes in forebrains analyzed by RT-qPCR (normalized to *β-actin* levels, relative to expression in wild-type). Each dot represents an individual embryo. *Pax9* and *Hoxb5* expression were below detection. Representative genes are shown for the following chromatin states: (A) H3K27me3, (B) H3K4me3/H3K27me3 and (C) unmodified.
(TIF)

Figure S11 Gene expression analysis in Jarid1b knockout embryos and adults. (A) Expression of *Otx2*, *Pax6* and *Sema5b* in heads of E12.5 embryos analyzed by RT-qPCR. Each dot represents an individual embryo. (B) Percent of neural stem cells (NSC) and neuronal progenitors (NP) in heads of E12.5 embryos analyzed by flow cytometry. (C) Expression in forebrains of newborn *Jarid1b* knockout pups that survived up to 2 hours after caesarean (“alive”) or died within 2 hours. (D) Expression in forebrains of adult male mice.
(TIF)

Figure S12 Model of the role of Jarid1b during mouse embryogenesis. Jarid1b regulates mouse development by protecting developmental genes from inappropriate acquisition of H3K4me3. In the absence of *Jarid1b*, neural master regulator genes like *Pax6* and *Otx2* are expressed at higher levels. Deletion of *Jarid1b* results in major neonatal lethality due to respiratory failure. Moreover, *Jarid1b* knockout embryos have several defects including disorganized cranial nerves, defects in eye development, skeletal transformations and increased incidences of morphological defects.
(TIF)

Figure S13 Survival of Jarid1b pups on a mixed C57BL/6/129 background. (A) Litter obtained from *Jarid1b* heterozygous parents (50% C57BL/6, 50% 129) immediately after caesarean delivery. Asteric indicates *Jarid1b* knockout pup. Note that the *Jarid1b* knockout pup has an open head (arrowhead). (B) Survival curve of *Jarid1b* wild-type (n = 16), heterozygote (n = 34) and knockout (n = 15) pups during the first day after caesarean delivery. (C) Body weight of pups immediately after delivery. (D) Examples of E17.5 embryos with indicated genotypes. (E) Frequency of *Jarid1b* wild-type (n = 25), heterozygote (n = 47) and knockout (n = 31) pups and embryos that develop exencephaly or other defects. (F) Percent of newborn *Jarid1b* mice that reacted to a tail pinch stimulus with a strong (2), weak (1) or no response (0). Scale bars, 1cm.
(TIF)

Table S1 Primer sequences.
(XLSX)
