# Supplementary figures and images for: The Histone Demethylase Jarid1b Ensures Faithful Mouse Development by Protecting Developmental Genes from Aberrant H3K4me3
Source: PLoS Genet. 2013 Apr 18;9(4):e1003461. doi: 10.1371/journal.pgen.1003461 (PMC3630093; doi:10.1371/journal.pgen.1003461)

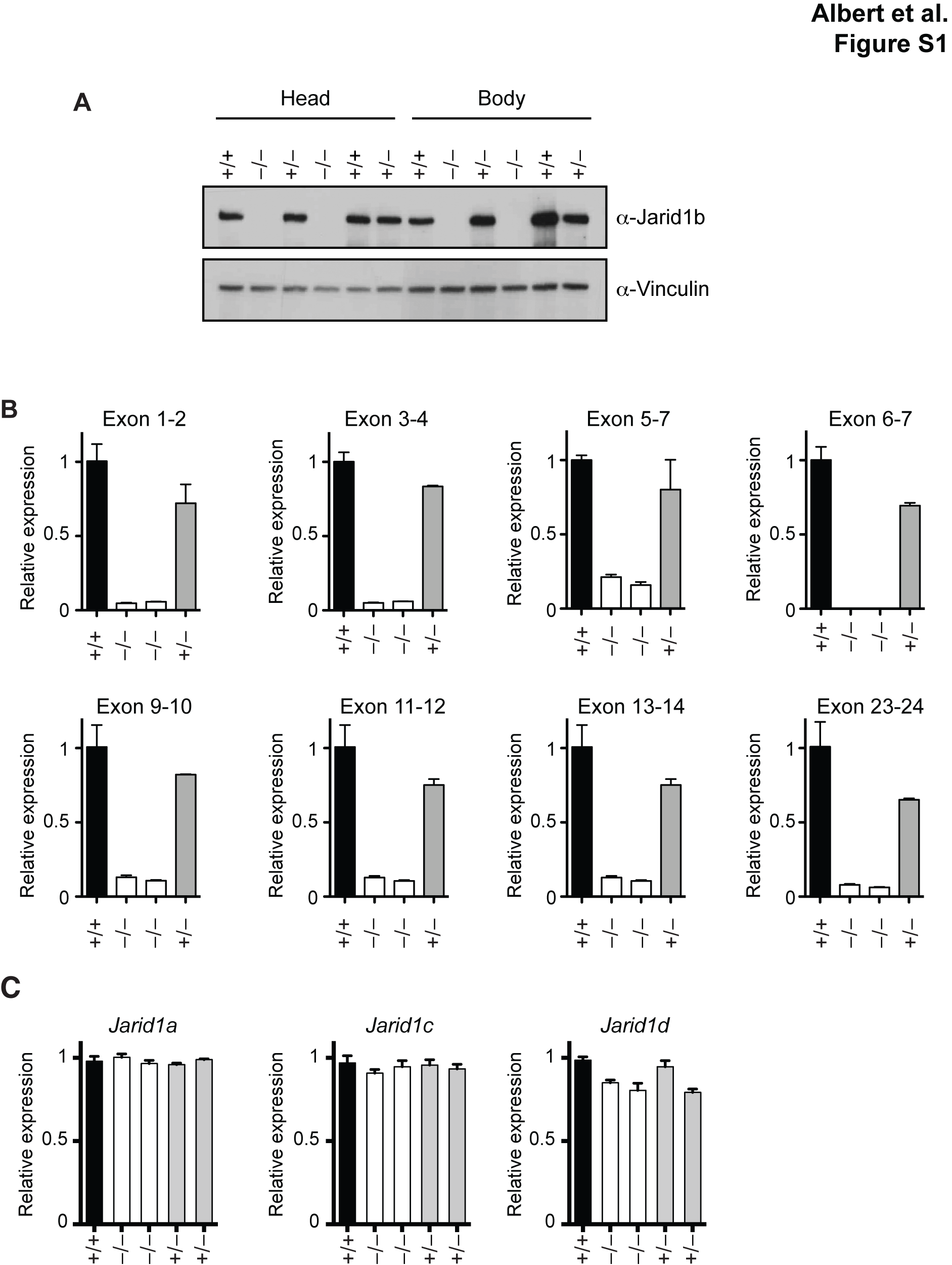

Supplement: Figure S1 — Expression of Jarid1b is lost in knockout embryos. (A) Immunoblots of E12.5 Jarid1b embryos divided into head and body probed for Jarid1b and Vinculin (loading control). (B) Expression of Jarid1b in the head of wild-type (+/+), knockout (−/−) and heterozygous (+/−) embryos (E12.5) determined by quantitative RT-PCR (normalized to β-actin levels). Primers were designed to cover exons encoding the following functional domains: JmjN (E1–2), ARID (E3 and E3–4), JmjC (E11–12 and E13–14) and PHD2 (E23–24). (C) Expression of Jarid1a, Jarid1c (located on the x chromosome) and Jarid1d (located on the y chromosome) in heads of male E12.5 embryos. (TIF) [file pgen.1003461.s001.tif]

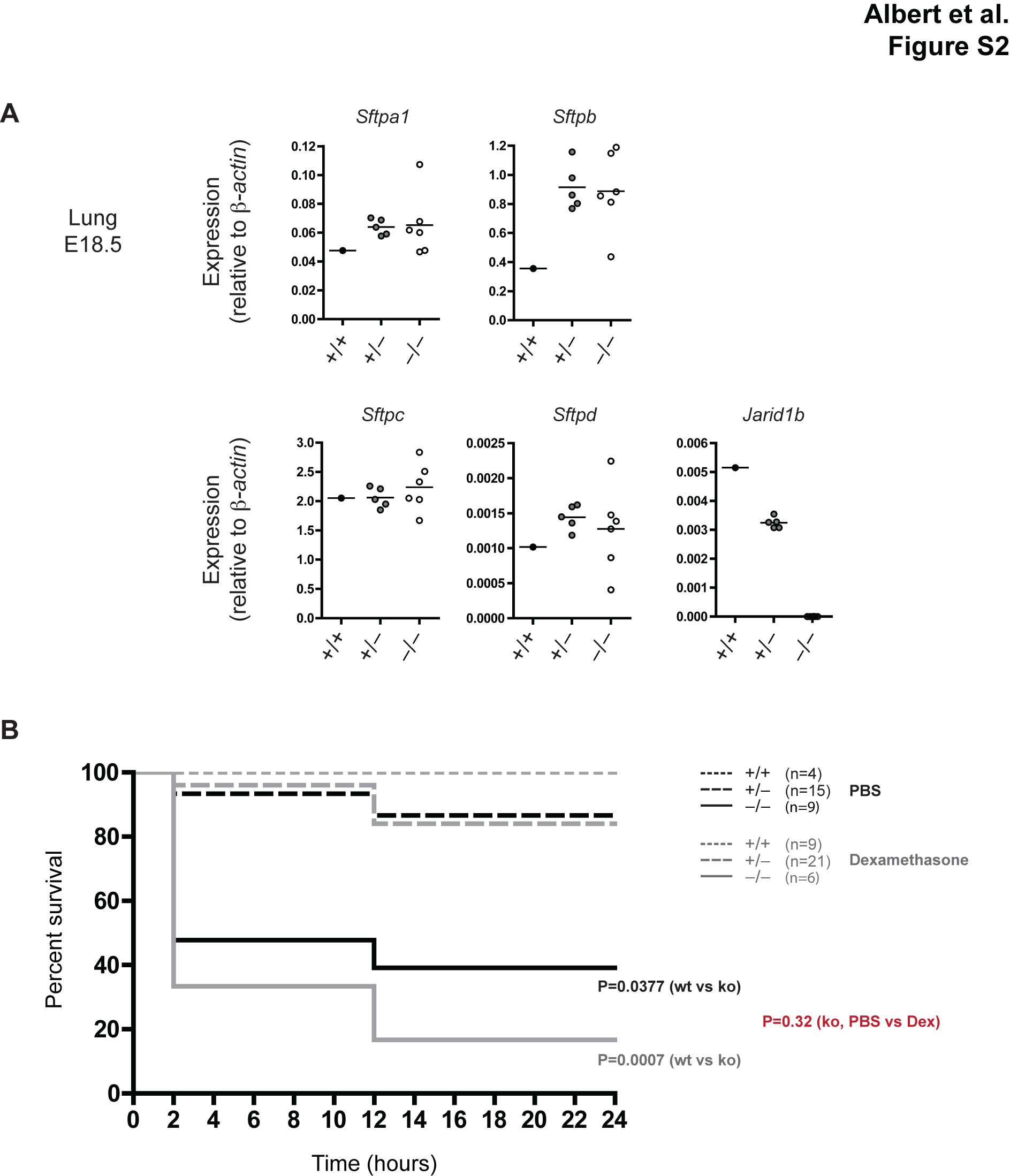

Supplement: Figure S2 — Lung maturation in Jarid1b knockouts. (A) Expression of surfactant proteins in lungs of E18.5 Jarid1b knockout embryos analyzed by RT-qPCR. Each dot represents an individual embryo. (B) Survival curve of Jarid1b wild-type, heterozygous and knockout pups treated with saline (PBS) or dexamethasone prior to cesarean delivery to induce lung maturation. Note that the curves for wild-type PBS and wild-type dexamethasone treatment are identical. (TIF) [file pgen.1003461.s002.tif]

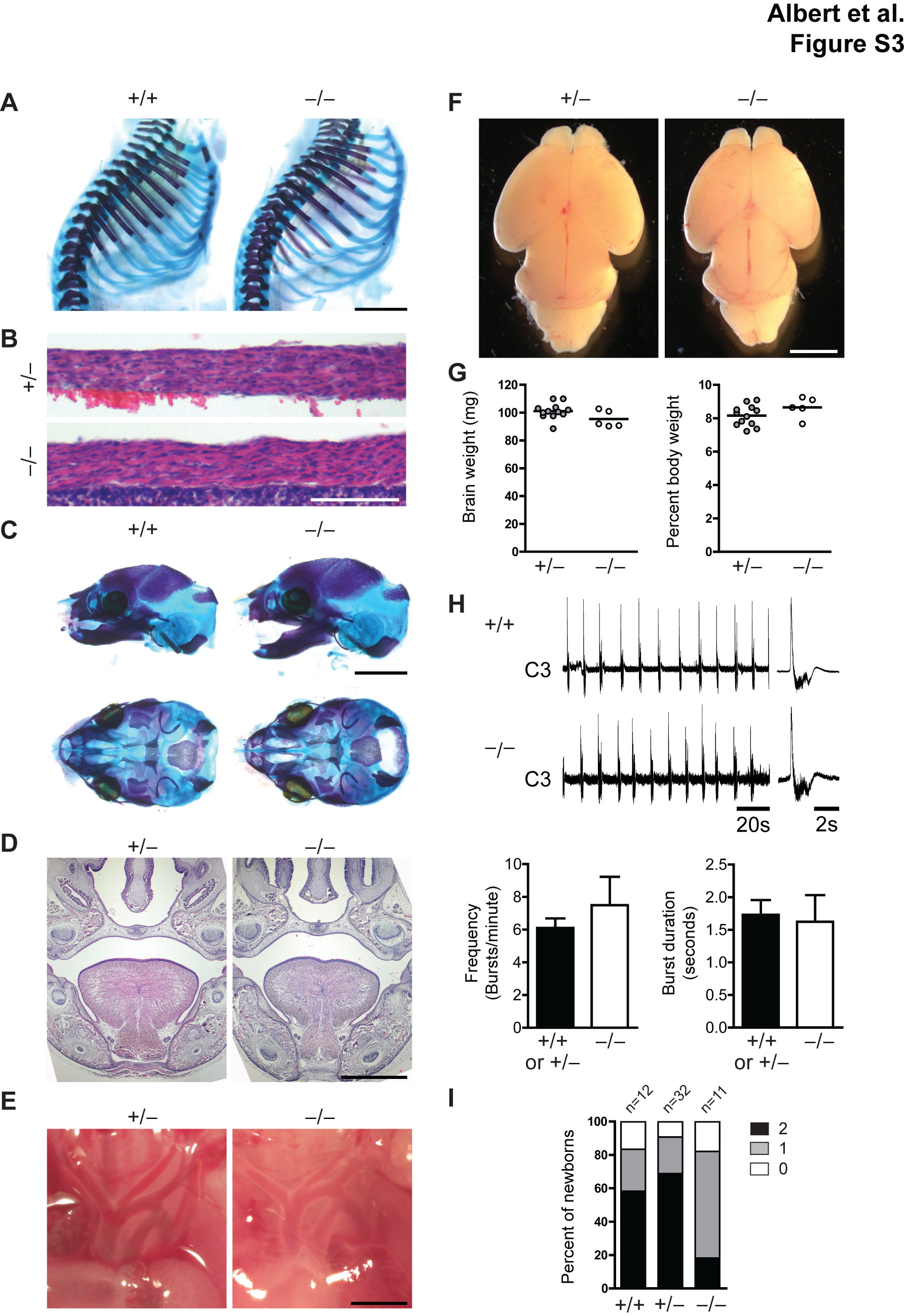

Supplement: Figure S3 — Other organs required for neonatal survival are grossly normal in Jarid1b knockouts. (A) Skeletal preparations of E17.5 Jarid1b embryos stained with Alcian blue (cartilage) and Alizarin red (bone). Shown is a lateral view of the rib cage. Scale bar, 2 mm. (B) Hematoxylin and eosin staining of paraffin-embedded diaphragms of E18.5 embryos cut sagittally. Scale bar, 50 µm. (C) Preparation of the skull of E17.5 embryos (top: side view; bottom: view from below). Scale bar, 2 mm. (D) Hematoxylin and eosin staining of coronal sections of the palatal region of E18.5 embryos. Scale bar, 0.5 mm. (E) Dissection of E18.5 embryos visualizing the cardiovascular system. Scale bar, 1 mm. (F) Brains of newborn mice (P0). Scale bar, 2 mm. (G) Weight of Jarid1b heterozygote and knockout brains at P0 in mg (left) or as percent body weight (right). (H) Spontaneous C3 nerve-activity in brainstem-spinal cord preparations from E18.5 embryos. Cycle-triggered averages of the inspiratory bursts are shown to the right. Below: Frequency and duration of the spontaneous nerve-activity in pooled wild-type/heterozygous (n = 5) and knockout (n = 6) Jarid1b embryos. (I) Percent of newborn Jarid1b mice that reacted to a tail pinch stimulus with a strong (2), weak (1) or no response (0). (TIF) [file pgen.1003461.s003.tif]

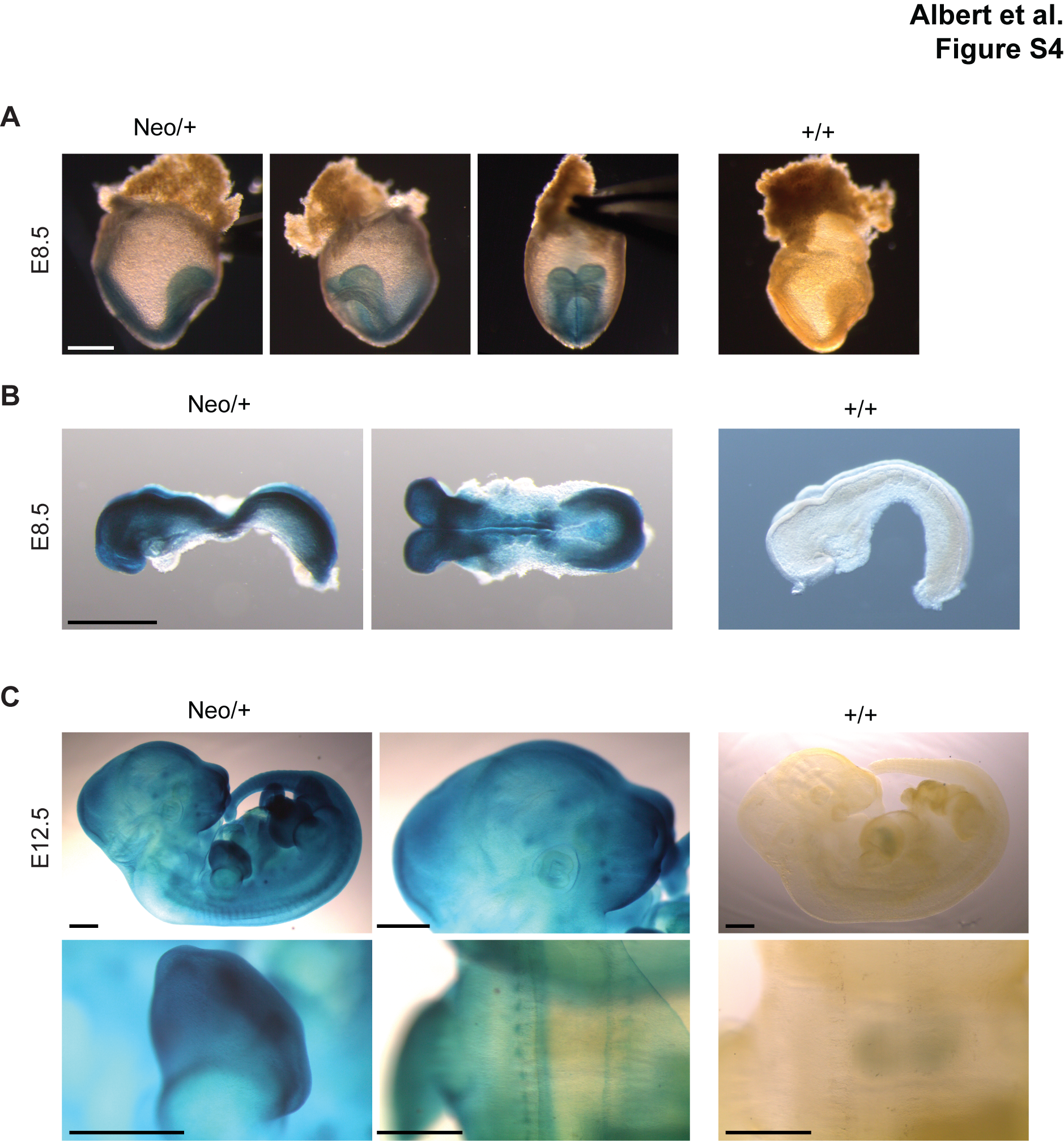

Supplement: Figure S4 — Jarid1b is widely expressed during embryogenesis. Staining for β-galactosidase in Jarid1b Neo/+ E8.5 embryos (A: embryo embedded in extraembryonic tissue; B: embryo dissected free of extraembryonic tissue; Scale bars, 0.5 mm) and E12.5 embryos (C; Scale bars, 1 mm) representing Jarid1b expression. Staining of Jarid1b +/+ embryos served as negative control. (TIF) [file pgen.1003461.s004.tif]

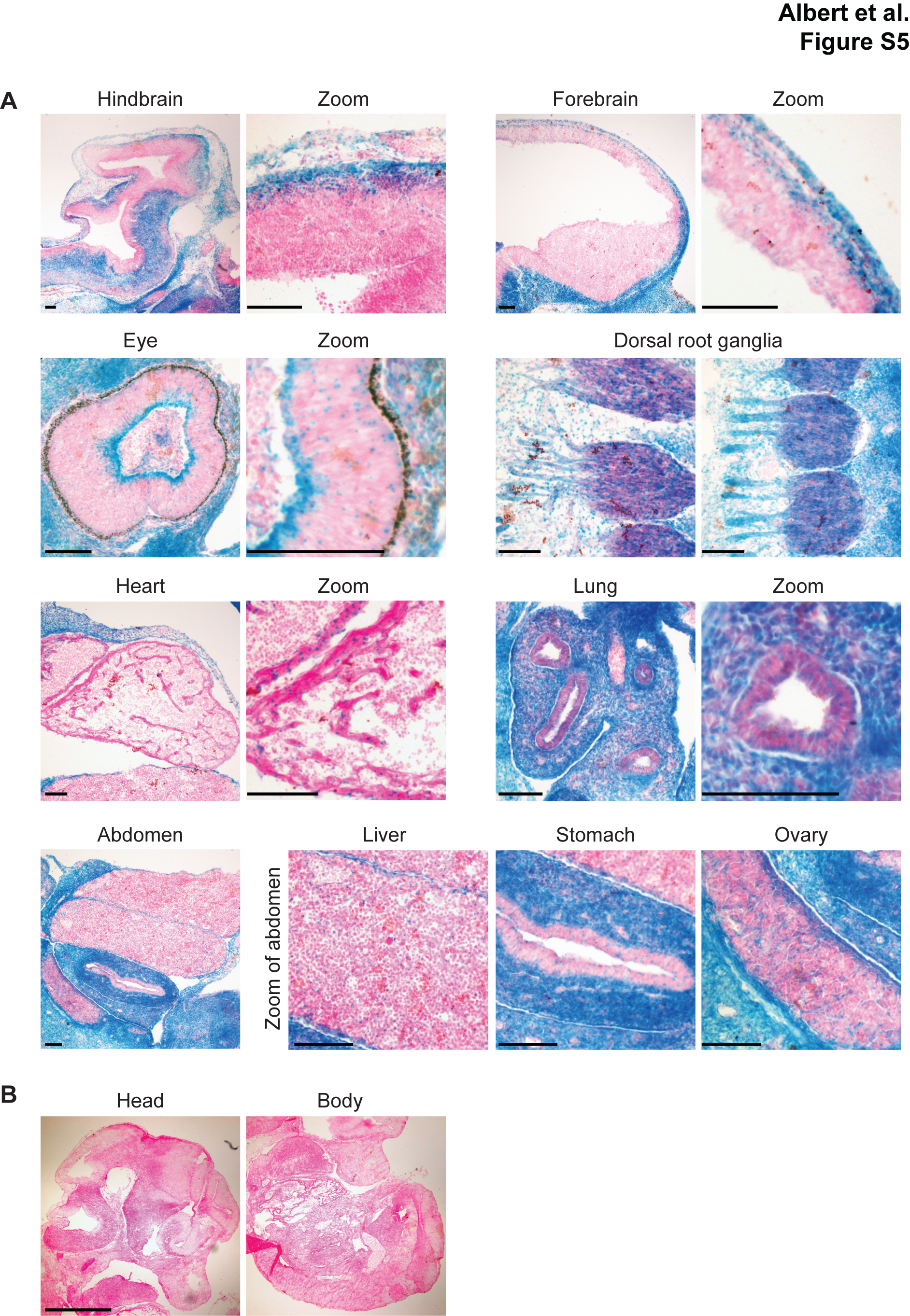

Supplement: Figure S5 — Jarid1b expression in E12.5 embryos. (A) Staining for β-galactosidase on sagittaly cut 8 µm cryo-sections of E12.5 Jarid1b Neo/+ embryos representing Jarid1b expression. Scale bars, 100 µm. (B) Staining of Jarid1b +/+ E12.5 embryos (negative control). Scale bar, 1 mm. Top: anterior; left: dorsal. (TIF) [file pgen.1003461.s005.tif]

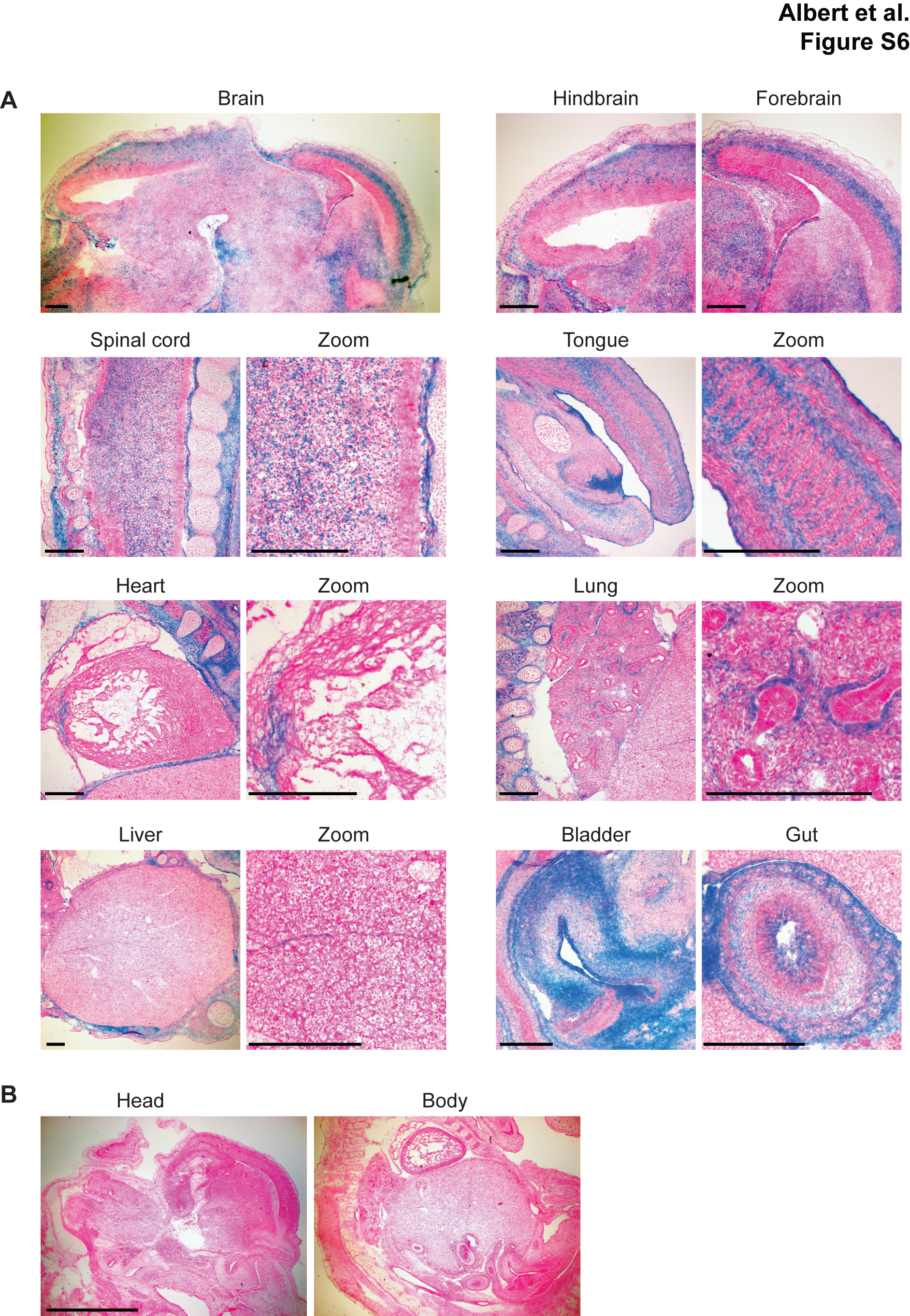

Supplement: Figure S6 — Jarid1b expression in E14.5 embryos. (A) Staining for β-galactosidase on sagittal sections of E14.5 Jarid1b Neo/+ embryos. Scale bars, 250 µm. (B) Staining of Jarid1b +/+ E14.5 embryos (negative control). Scale bar, 1 mm. Top: anterior; left: dorsal. (TIF) [file pgen.1003461.s006.tif]

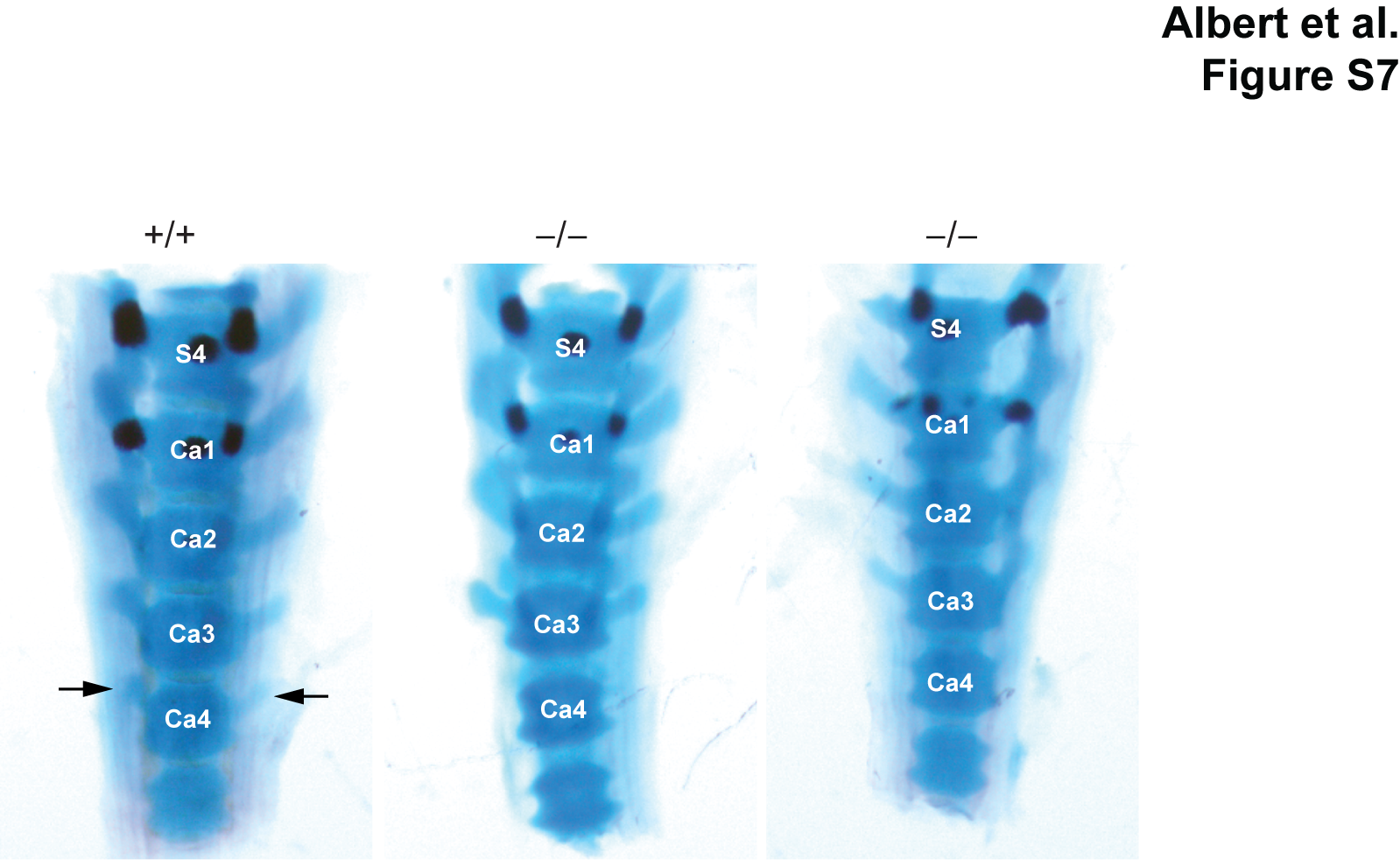

Supplement: Figure S7 — Skeletal transformations in Jarid1b knockout embryos. Skeletal preparations of E17.5 Jarid1b embryos stained with Alcian blue (cartilage) and Alizarin red (bone). Shown is a ventral view of the sacral-caudal region. Note that the 34th vertebra (Ca4 in wild-type) lacks transverse processes in Jarid1b knockouts. Arrows indicate transverse processes in wild-type. S: sacral; Ca: caudal. (TIF) [file pgen.1003461.s007.tif]

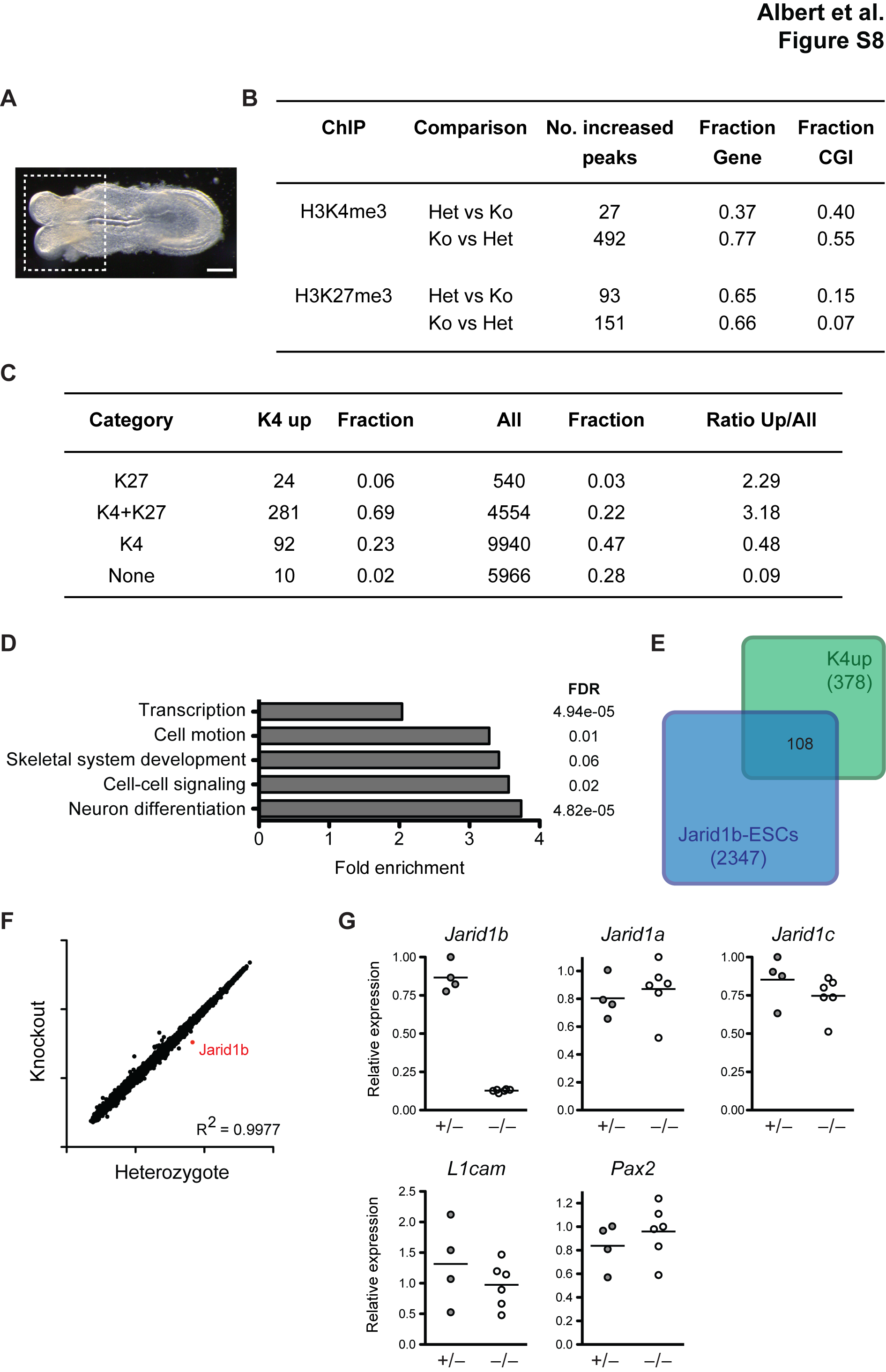

Supplement: Figure S8 — Characterization of loci with increased H3K4me3 in early Jarid1b knockout embryos. (A) Head region (boxed) of E8.5 embryos (3–8 somites) was used for ChIP. Scale bar, 0.2 mm. (B) Summary of ChIP-seq comparison of H3K4me3 and H3K27me3 between Jarid1b heterozygous and knockout embryos (E8.5). The number of peaks with increased histone methylation is shown as well as the fraction of peaks that overlaps with genes or CpG islands (CGI). (C) Number of genes with H3K27me3, H3K4me3/H3K27me3, H3K4me3 and unmodified genes among genes with elevated H3K4me3 levels (K4 up) compared to all genes. (D) Gene ontology analysis of genes with elevated H3K4me3 levels using the BP-FAT category in the DAVID software. (E) Venn diagram showing the overlap between genes bound by Jarid1b in ESCs and genes showing elevated levels of H3K4me3 in E8.5 Jarid1b knockout embryos. (F) Scatter blot of global gene expression microarrays comparing Jarid1b heterozygous and knockout E8.5 embryos (head regions). Red dot represents Jarid1b, the only gene that changes more than 1.5-fold. The correlation coefficient R2 is indicated. Values represent averages of three biological replicates. (G) Expression of Jarid1b, Jarid1a, Jarid1c, L1cam and Pax2 in E8.5 embryos was determined by RT-qPCR (normalized to β-actin). Each dot represents an individual embryo. (TIF) [file pgen.1003461.s008.tif]

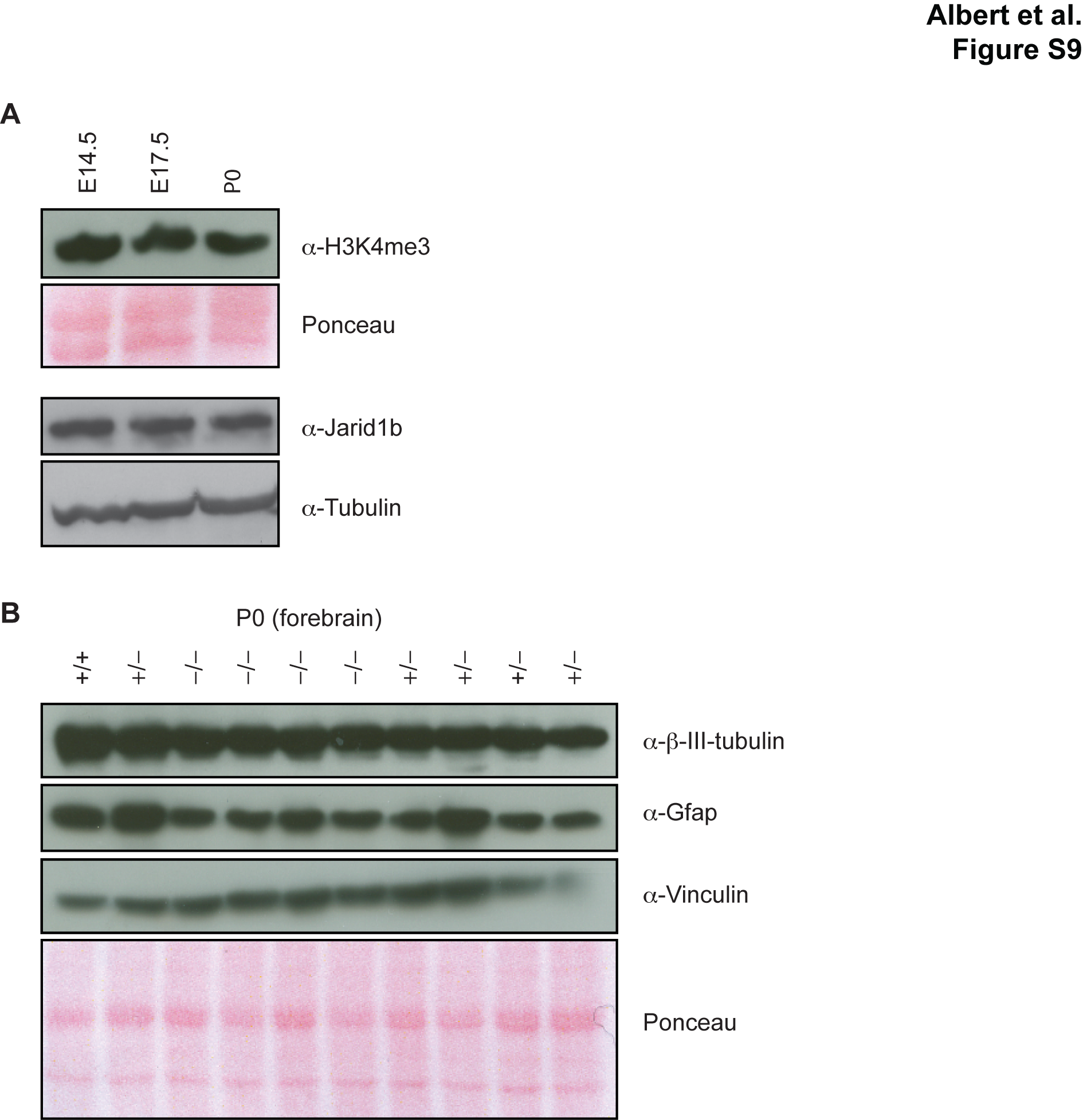

Supplement: Figure S9 — H3K4me3 during brain development. (A) Immunoblots for H3K4me3 and Jarid1b from heads of wild-type E14.5 and E17.5 embryos and wild-type P0 brains. (B) Immunoblots for markers of neurons (β-III-tubulin) and astrocytes (Gfap) of Jarid1b wild-type, heterozygous and knockout P0 brains. Tubulin and Ponceau serve as loading controls. (TIF) [file pgen.1003461.s009.tif]

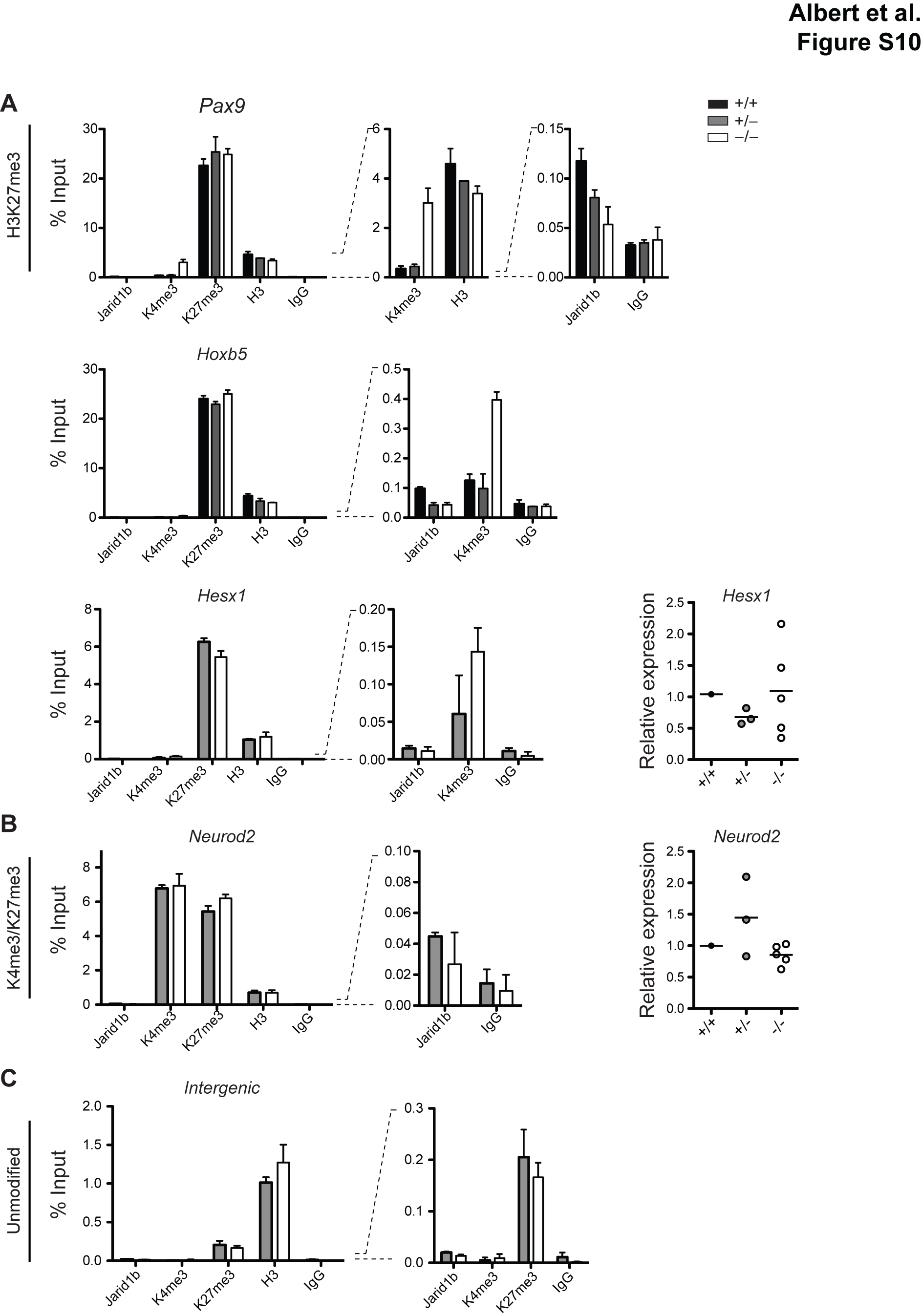

Supplement: Figure S10 — H3K4me3 levels at genes and gene expression in Jarid1b knockout brains after birth. (A–C) Left: ChIP-qPCR for Jarid1b, H3K4me3, H3K27me3, H3 and IgG in forebrains of P0 pups. Error bars represent S.D. of three PCR amplifications. Right: Expression of indicated genes in forebrains analyzed by RT-qPCR (normalized to β-actin levels, relative to expression in wild-type). Each dot represents an individual embryo. Pax9 and Hoxb5 expression were below detection. Representative genes are shown for the following chromatin states: (A) H3K27me3, (B) H3K4me3/H3K27me3 and (C) unmodified. (TIF) [file pgen.1003461.s010.tif]

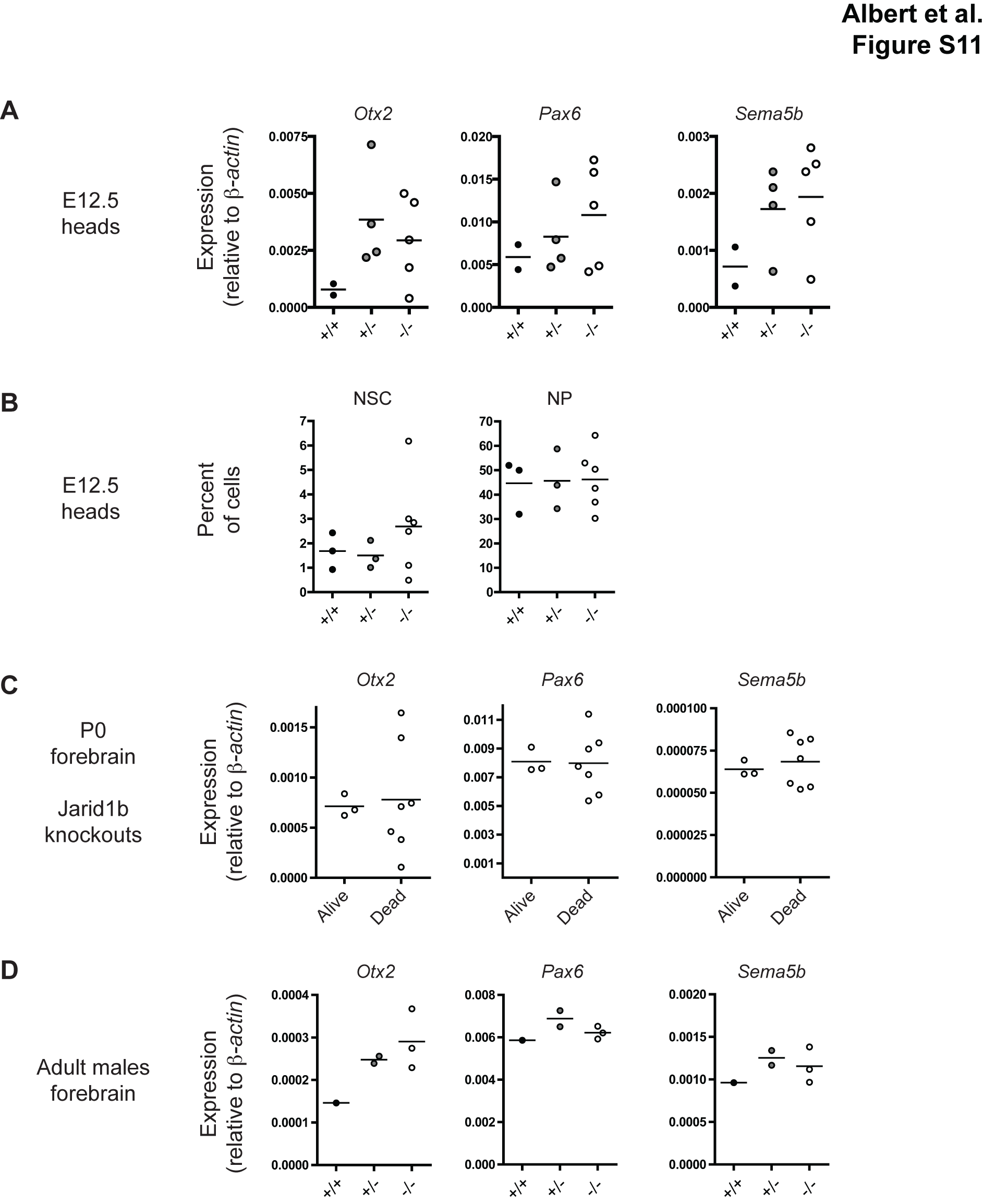

Supplement: Figure S11 — Gene expression analysis in Jarid1b knockout embryos and adults. (A) Expression of Otx2, Pax6 and Sema5b in heads of E12.5 embryos analyzed by RT-qPCR. Each dot represents an individual embryo. (B) Percent of neural stem cells (NSC) and neuronal progenitors (NP) in heads of E12.5 embryos analyzed by flow cytometry. (C) Expression in forebrains of newborn Jarid1b knockout pups that survived up to 2 hours after caesarean (“alive”) or died within 2 hours. (D) Expression in forebrains of adult male mice. (TIF) [file pgen.1003461.s011.tif]

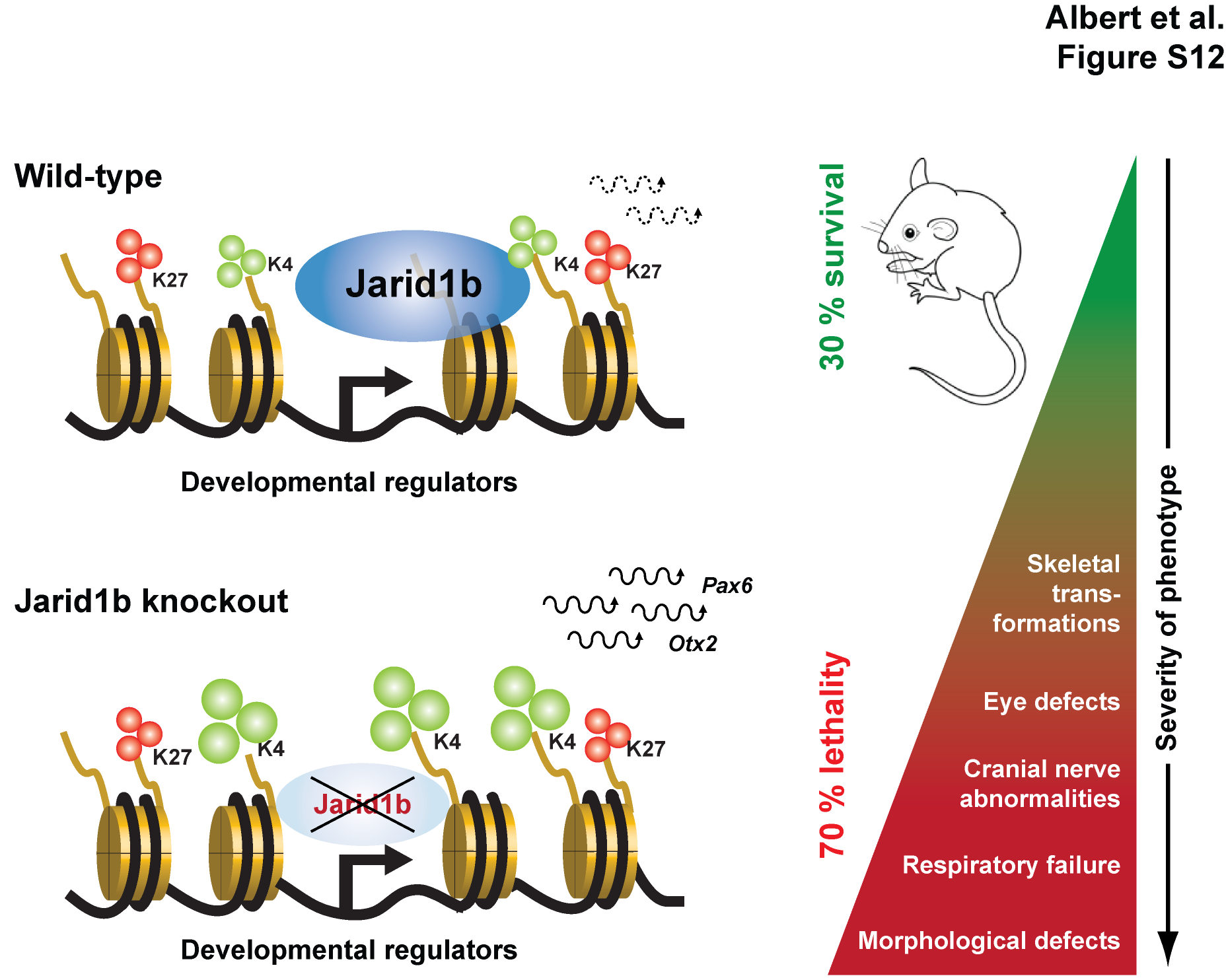

Supplement: Figure S12 — Model of the role of Jarid1b during mouse embryogenesis. Jarid1b regulates mouse development by protecting developmental genes from inappropriate acquisition of H3K4me3. In the absence of Jarid1b, neural master regulator genes like Pax6 and Otx2 are expressed at higher levels. Deletion of Jarid1b results in major neonatal lethality due to respiratory failure. Moreover, Jarid1b knockout embryos have several defects including disorganized cranial nerves, defects in eye development, skeletal transformations and increased incidences of morphological defects. (TIF) [file pgen.1003461.s012.tif]

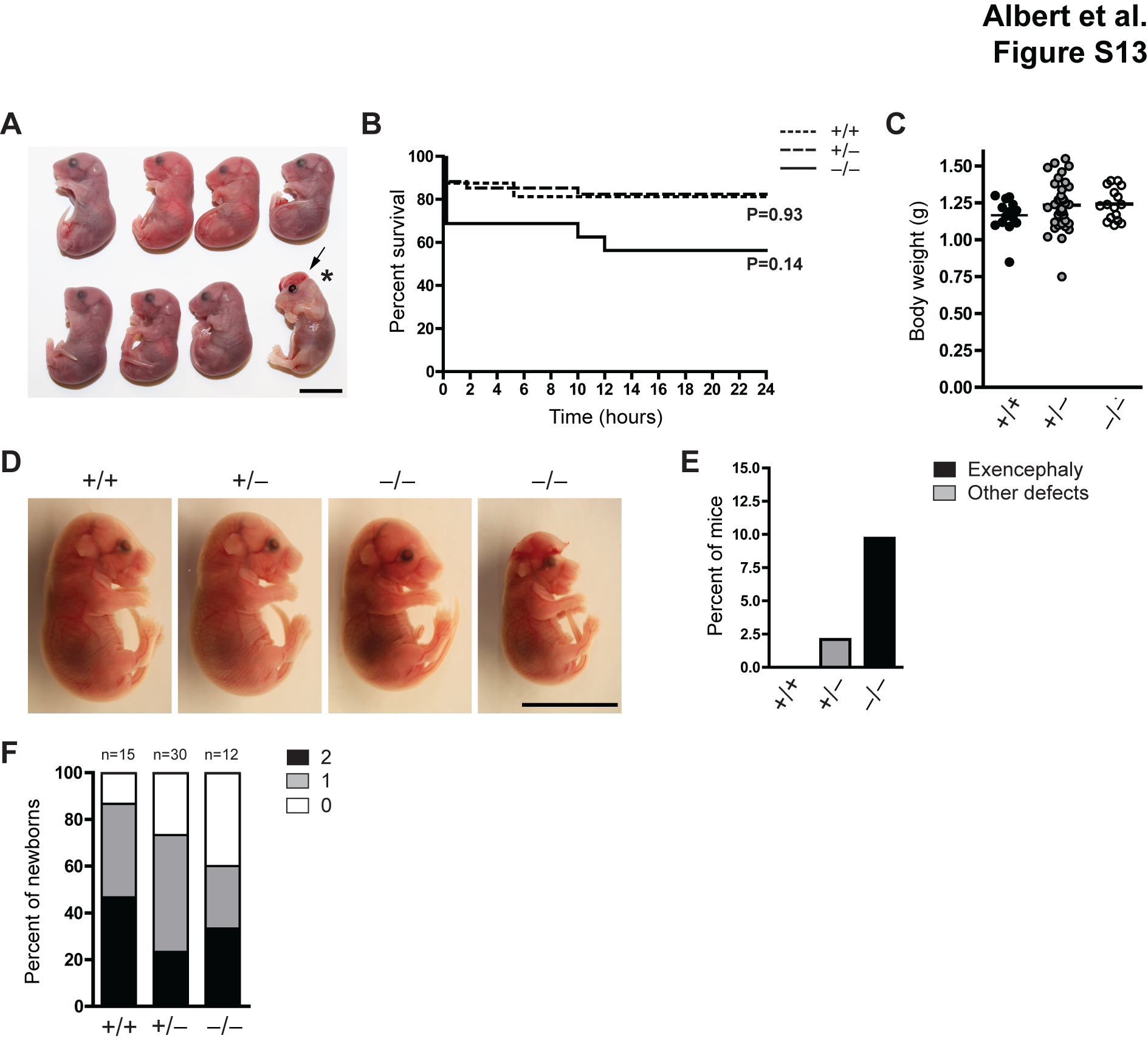

Supplement: Figure S13 — Survival of Jarid1b pups on a mixed C57BL/6/129 background. (A) Litter obtained from Jarid1b heterozygous parents (50% C57BL/6, 50% 129) immediately after caesarean delivery. Asteric indicates Jarid1b knockout pup. Note that the Jarid1b knockout pup has an open head (arrowhead). (B) Survival curve of Jarid1b wild-type (n = 16), heterozygote (n = 34) and knockout (n = 15) pups during the first day after caesarean delivery. (C) Body weight of pups immediately after delivery. (D) Examples of E17.5 embryos with indicated genotypes. (E) Frequency of Jarid1b wild-type (n = 25), heterozygote (n = 47) and knockout (n = 31) pups and embryos that develop exencephaly or other defects. (F) Percent of newborn Jarid1b mice that reacted to a tail pinch stimulus with a strong (2), weak (1) or no response (0). Scale bars, 1cm. (TIF) [file pgen.1003461.s013.tif]
